# Tailored parameter optimization methods for ordinary differential equation models with steady-state constraints

**DOI:** 10.1186/s12918-016-0319-7

**Published:** 2016-08-22

**Authors:** Anna Fiedler, Sebastian Raeth, Fabian J. Theis, Angelika Hausser, Jan Hasenauer

**Affiliations:** 1 Institute of Computational Biology, Helmholtz Zentrum München, Ingolstädter Landstraße 1, Neuherberg, 85764 Germany; 2 Chair of Mathematical Modeling of Biological Systems, Center for Mathematics, Technische Universität München, Boltzmannstraße 3, Garching, 85748 Germany; 3 Stuttgart Research Center Systems Biology (SRCSB), University of Stuttgart, Nobelstr. 15, Stuttgart, 70569 Germany

**Keywords:** Parameter optimization, Differential equation, Steady state, Perturbation experiments

## Abstract

**Background:**

Ordinary differential equation (ODE) models are widely used to describe (bio-)chemical and biological processes. To enhance the predictive power of these models, their unknown parameters are estimated from experimental data. These experimental data are mostly collected in perturbation experiments, in which the processes are pushed out of steady state by applying a stimulus. The information that the initial condition is a steady state of the unperturbed process provides valuable information, as it restricts the dynamics of the process and thereby the parameters. However, implementing steady-state constraints in the optimization often results in convergence problems.

**Results:**

In this manuscript, we propose two new methods for solving optimization problems with steady-state constraints. The first method exploits ideas from optimization algorithms on manifolds and introduces a retraction operator, essentially reducing the dimension of the optimization problem. The second method is based on the continuous analogue of the optimization problem. This continuous analogue is an ODE whose equilibrium points are the optima of the constrained optimization problem. This equivalence enables the use of adaptive numerical methods for solving optimization problems with steady-state constraints. Both methods are tailored to the problem structure and exploit the local geometry of the steady-state manifold and its stability properties. A parameterization of the steady-state manifold is not required.

The efficiency and reliability of the proposed methods is evaluated using one toy example and two applications. The first application example uses published data while the second uses a novel dataset for Raf/MEK/ERK signaling. The proposed methods demonstrated better convergence properties than state-of-the-art methods employed in systems and computational biology. Furthermore, the average computation time per converged start is significantly lower. In addition to the theoretical results, the analysis of the dataset for Raf/MEK/ERK signaling provides novel biological insights regarding the existence of feedback regulation.

**Conclusion:**

Many optimization problems considered in systems and computational biology are subject to steady-state constraints. While most optimization methods have convergence problems if these steady-state constraints are highly nonlinear, the methods presented recover the convergence properties of optimizers which can exploit an analytical expression for the parameter-dependent steady state. This renders them an excellent alternative to methods which are currently employed in systems and computational biology.

**Electronic supplementary material:**

The online version of this article (doi:10.1186/s12918-016-0319-7) contains supplementary material, which is available to authorized users.

## Background

Gene regulation, signal transduction, metabolism and many other biological processes are nowadays analyzed using mathematical models [1]. Mathematical models allow for the integration of available knowledge, providing mechanistic insights and an understanding of design principles [2]. The spectrum of employed modeling approaches ranges from qualitative Boolean models [3, 4] to quantitative stochastic spatial models [5]. In particular ODE models, such as reaction rate equations, are used on a range of spatial and temporal scales [6]. These models describe the temporal evolution of the concentration of biochemical species in cellular compartments as a function of initial conditions, parameters and stimuli.

ODE models are flexible and allow for the mechanistic description of a range of processes (see, e.g., [7–9] and references therein). Similar to other quantitative modeling approaches, ODE models rely on accurate values for initial conditions and parameters, e.g., binding affinities, synthesis and degradation rates. Initial conditions and parameters are often unknown and have to be inferred from experimental data [10].

**Fig. 1:**
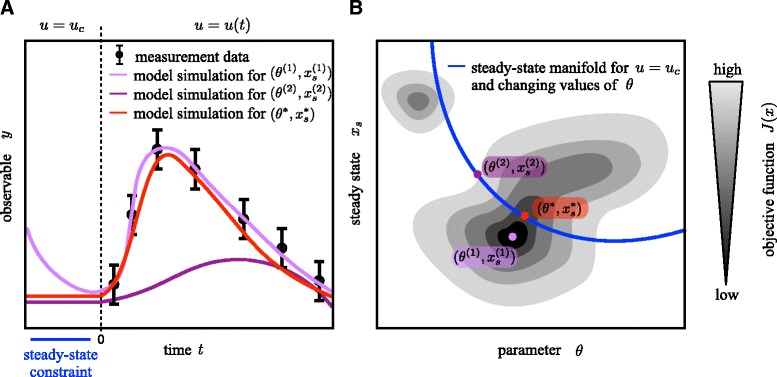
Schematic illustration of optimization problem with steady-state constraint. **a** Measurement data and simulations of the system for three different pairs of parameters and initial conditions: optimum of the unconstrained optimization problem $(\theta ^{(1)},x_{s}^{(1)})$; suboptimal point on the manifold $(\theta ^{(2)}, x_{s}^{(2)} = x_{s}(u_{0},\theta ^{(2)}))$; and optimum of the constrained optimization problem $(\theta ^{*}, x_{s}^{*} = x_{s}(u_{0},\theta ^{*}))$. The system is perturbed at time *t*=0 and should be in steady state for *t*<0. **b** Objective function landscape, steady-state manifold and pairs of parameters and initial conditions

In most studies, experimental data from *perturbation experiments* are used to infer these unknown parameters [11, 12]. In perturbation experiments, the response of the process to an external stimulus (also denoted as perturbation) is quantified, as illustrated in Fig. 1a. As the initial condition of the process corresponds to a stable steady state of the unperturbed system, perturbation experiments provide information about the stimulus response. Depending on the process and the input, the stimulus-induced changes might be transient or persistent. Commonly used stimuli are ligands, which bind to receptors and induce downstream signaling, small molecules, which diffuse across the cell membrane and change the cell state, and physical stimuli (e.g., heat, cold or force).

The estimation of the parameters of ODE models from data collected during perturbation experiments requires the solution of optimization problems. These optimization problems are in general nonlinear, non-convex and computationally demanding. This establishes the need for efficient and robust optimization methods [13, 14]. In the literature, multi-start local optimization methods [15] and global optimization methods [16, 17] have been employed. If the stationarity of the initial condition does not have to be enforced or if analytical expressions of the steady state are available, these methods mostly work well [15, 17]. However, neglecting steady-state constraints often results in a loss of information and potentially implausible predictions. Furthermore, an analytical expression of the parameter and input-dependent steady state is rarely available.

Steady-state constraints are nonlinear equality constraints, which restrict the solution space to a manifold of feasible points. Enforcing these equality constraints causes efficiency and convergence problems for standard optimization methods [18]. Deterministic local optimization methods have to move along the manifold, resulting in small step-sizes or stagnation. Stochastic local and global optimization methods are only allowed to propose update steps on the manifold, which is not possible in state-of-the-art toolboxes (see, e.g., [19, 20]). The elimination of the equality constraints using analytical expressions for the parameter-dependent steady states resolves these problems [18].

The first method to derive analytical expressions for steady states has been proposed by [21] for networks of enzyme-catalyzed reactions. This method was later implemented [22] and subsequently extended using results from graph theory [23, 24]. Furthermore, tailored methods for models with bilinear rate laws have been introduced [25]. For models with Michaelis-Menten and Hill-Type kinetics, py-substitution has been developed [26]. This method solves the steady-state constraint for a combination of states and parameters. An important recent extension ensured positivity of the solution [18]. Py-substitution and its extension are however only applicable if the model possesses sufficiently many degrees of freedom [18], which is difficult to assess a priori.

We propose two novel methods for solving optimization problems with a single or multiple steady-state constraints. These methods do not rely on an analytical expression for the steady state. Instead, the geometry of the steady-state manifold and the stability of the steady state are exploited.

The first method we introduce borrows ideas from optimization algorithms on matrix manifolds [27, 28]. These optimization algorithms employ retraction operators which map a point onto the manifold [28]. These retraction operators – usually analytical functions – facilitate an effective movement of optimizers on the manifold. Retraction operators are however problem-specific and their construction is usually non-trivial [28], which limits the application of established algorithms for optimization on manifolds. We exploit therefore a simulation-based retraction operator which exploits the stability of the steady state. This retraction operator can be used within state-of-the-art optimizers to eliminate the equality constraint and reduce the problem dimension. As the method uses both, a simulation-based retraction operator and a state-of-the-art optimizer, we will in the following refer to it as a hybrid optimization method.

The second method uses continuous analogues of local optimizers [29]. Continuous analogues are dynamical systems whose trajectories converge to a locally optimal point of an optimization problem. These dynamical systems often possess a larger basin of attraction [30] and can be solved using sophisticated numerical methods. This promises more robust convergence than simple step-size controls used in existing optimization methods. Continuous analogues have been constructed for a series of linear and nonlinear problems [30–32]. We introduce continuous gradient descent and Newton-Raphson methods for solving optimization problems with steady-state constraints. The manifold is stabilized using a continuous retraction derived from the model. This method is purely simulation-based and will therefore be referred to as a simulation-based optimization method.

The proposed optimization methods are illustrated using a simulation example. This is followed by a rigorous evaluation of the methods and comparison with alternative methods. To this end we consider two applications: NGF-induced activation of ERK in primary sensory neurons; and Raf/MEK/ERK signaling in HeLa cells. Using novel data for the second application, new insights into Raf/MEK/ERK signaling upon release from S-phase arrest are discovered.

## Methods

In this section we introduce the model class and the optimization problem. The differential geometry of the steady-state manifold is described and two optimization methods exploiting this geometry are introduced. The properties of these methods along with their implementation are discussed.

The optimization methods are used in the *Results* section to study Raf/MEK/ERK signaling in HeLa cells after release from S-phase arrest. The biological materials and the setups used to study this process experimentally are described below.

### Mathematical modeling of perturbation experiments

In this manuscript we consider ODE models of biochemical reaction networks. ODE models are quite general and allow for the description of many gene regulation, signal transduction and metabolic processes [1]. Mathematically, ODE models are commonly written as

1 $$ \begin{aligned} \frac{dx}{dt} &= f(x,\theta,u), \quad x(0) = x_{0}(\theta)\\ y &= h(x,\theta,u), \end{aligned}  $$

with states $x(t) \in \mathbb {R}^{n_{x}}$, observables $y(t) \in \mathbb {R}^{n_{y}}$, parameters $\theta \in \mathbb {R}^{n_{\theta }}$ and inputs $u(t) \in \mathbb {R}^{n_{u}}$. The states *x*(*t*) are the concentrations of biochemical species at time *t*. The observables *y*(*t*) are the values of measurable quantities. The parameters *θ* are biochemical reaction rates, total abundances of biochemical species (in the presence of conservation relations) and experimental parameters (e.g. scaling and offset). The inputs *u* encode the experimental conditions applied to the system, e.g., extracellular concentration of ligands. To ensure existence and uniqueness of the solution of (1), the vector field $f:\mathbb {R}^{n_{x}} \times \mathbb {R}^{n_{\theta }} \times \mathbb {R}^{n_{u}} \to \mathbb {R}^{n_{x}}$ is assumed to be Lipschitz continuous. The mapping $h:\mathbb {R}^{n_{x}} \times \mathbb {R}^{n_{\theta }} \times \mathbb {R}^{n_{u}} \to \mathbb {R}^{n_{y}}$ describes the observation process and the mapping $x_{0}:\mathbb {R}^{n_{\theta }} \to \mathbb {R}^{n_{x}}$ provides the initial conditions.

The dynamics of model (1) are probed using perturbation experiments, *e*=1,…,*E*, with inputs *u*^*e*^. The initial condition *x*_0_(*θ*) for an experiment condition is the steady state ${x_{s}^{e}}$ for a control condition ($u = {u_{c}^{e}}$). This steady state ${x_{s}^{e}}$ is parameter-, and input-dependent and fulfills the steady-state constraint,

2 $$ 0 = f\left({x_{s}^{e}},\theta,{u_{c}^{e}}\right).   $$

The stability of ${x_{s}^{e}}$ can be assessed using Lyapunov theory [33]. We denote the collection of all parameter-state pairs (*θ*,*x*_0_) which fulfill the steady-state constraint (2) for a given input *u* as the manifold of steady states. For simplicity, we assume that (2) possesses a unique, exponentially stable steady state for every combination of parameters *θ* and inputs *u*. In this case, there exists a function $x_{s}: \mathbb {R}^{n_{\theta }} \times \mathbb {R}^{n_{u}} \rightarrow \mathbb {R}^{n_{x}}$ which maps the parameters to the corresponding steady state, i.e., *x*(0)=*x*_*s*_(*θ*,*u*) (see Fig. 1b). An analytical expression of the function *x*_*s*_(*θ*,*u*) is usually not available.

Perturbation experiments provide measurement data,

3 $$ \mathcal{D} = \left\{ \left\{ \left(t_{j},\left\{\bar{y}_{ij}^{e}\right\}_{i=1}^{n_{y}} \right) \right\}_{j=1}^{N} \right\}_{e=1}^{E}.   $$

The observable is indexed by *i*, the time point is indexed by *j* and the experimental condition is indexed by *e*. The measurements are noise-corrupted,

4 $$ \bar{y}_{ij}^{e} = {y_{i}^{e}}\left(t_{j},\theta,{x_{s}^{e}}\right) + \epsilon_{ij}^{e},  $$

with $y^{e}\left (t,\theta,{x_{s}^{e}}\right)$ denoting the solution of (1) for input *u*=*u*^*e*^, parameters *θ* and initial condition ${x_{s}^{e}}$ at time *t*. The measurement noise $\epsilon _{ij}^{e}$ is assumed to be normally distributed, $\epsilon _{ij}^{e} \sim \mathcal {N}\left (0,\left (\sigma _{ij}^{e}\right)^{2}\right)$, the methods presented in the following are however not limited to this case.

### Parameter estimation

In this study we employ maximum likelihood (ML) estimation to determine the unknown model parameters *θ* and steady states ${x_{s}^{1}},\ldots,{x_{s}^{E}}$ from the experimental data $\mathcal {D}$. In accordance with the noise distribution, the likelihood function

5 $$ \begin{aligned} &p\left(\mathcal{D}|\theta,{x_{s}^{1}},\ldots,{x_{s}^{E}}\right) := \\ &\prod_{e = 1}^{E} \prod_{j = 1}^{N} \prod_{i = 1}^{n_{y}} \frac{1}{\sqrt{2 \pi} \sigma_{ij}^{e}} \exp\left\{ - \frac{1}{2}\left(\frac{\bar{y}_{ij}^{e} - {y_{i}^{e}}(t_{j},\theta,{x_{s}^{e}})}{\sigma_{ij}^{e}}\right)^{2} \right\} \end{aligned}  $$

is used. This likelihood function depends on *θ* and ${x_{s}^{1}},\ldots,{x_{s}^{E}}$, variables which are coupled via the steady-state constraint (2).

The ML estimates for parameters and initial conditions, $\hat \theta $ and $\left \{\hat {x}_{s}^{e}\right \}_{e=1}^{E}$, are obtained by maximizing the likelihood function (5) subject to the steady-state constraint (2). To improve the numerical evaluation and the optimizer convergence, this maximization problem is reformulated to the equivalent minimization problem

6 $$ \begin{aligned} \min_{\theta,{x_{s}^{1}},\ldots,x_{s}^{E}} &\; J\left(\theta,{x_{s}^{1}},\ldots,x_{s}^{E}\right) :=\\ & \frac{1}{2} \sum\limits_{e=1}^{E} \sum\limits_{j=1}^{N} \sum\limits_{i = 1}^{n_{y}} \left(\frac{\bar{y}_{i}^{e}\left(t_{j}\right) - {y_{i}^{e}}\left(t_{j},\theta,{x_{s}^{e}}\right)}{\sigma_{ij}^{e}}\right)^{2}\\ \mathrm{s.t.} \, &\; 0 = f\left({x_{s}^{e}},\theta,{u_{c}^{e}}\right), \quad e = 1,\ldots,E \end{aligned}  $$

in which the objective function denotes the negative log-likelihood function, $J\!\!\left (\!\theta \!,{x_{s}^{1}},\ldots,{x_{s}^{E}}\right) \,=\, -\! \log p\!\left (\!\mathcal {D}|\theta \!,{x_{s}^{1}},\ldots,{x_{s}^{E}}\right)$. The solution of (6) provides parameter-state pairs $\left (\hat \theta,\hat {x}_{s}^{e}\right)$ on the steady-states manifold which maximize the likelihood function (5). For these pairs it holds that $\hat {x}_{s}^{e} = x_{s}\left (\hat \theta,{u_{c}^{e}}\right)$.

In general, optimization problem (6) is nonlinear and possesses local minima. To compute the optimum of (6), we employ multi-start local optimization. This approach proved to be efficient in a variety of related problems (see, e.g., [15, 34]). Furthermore, sophisticated local optimizers allow for the consideration of nonlinear equality constraints (see [35] and references therein). The consideration of nonlinear equality constraints is not possible for most evolutionary and genetic algorithms [36], particle swarm optimizers [37], simulated annealing [38] and hybrid optimizers [19, 39]. Alternative methods are metaheuristics which combine ideas from local and global optimization methods [40], facilitating the analysis of optimization problems with nonlinear constraints and multiple local minima.

The performance of multi-start local optimization depends on the local optimization method. In this study four alternative methods are considered, two established methods:

- *Unconstrained optimization method:* An analytical expression of the steady state as a function of the parameter, *x*_*s*_(*θ*), is used to eliminate the constraint and *x*_0_ from optimization problem (6). This yields the reduced optimization problem
  7 $$ \begin{aligned} \min_{\theta} &\; J\left(\theta,x_{s}\left(\theta,{u_{c}^{1}}\right),\ldots,x_{s}\left(\theta,{u_{c}^{E}}\right)\right) \end{aligned}   $$
  which does not possess any equality constraints. While this method is rarely applicable – analytical expressions for the *x*_*s*_(*θ*) are difficult to compute – it provides a gold standard.
- *Constrained optimization method:* An interior point optimization method is used to solve the optimization problem (6). This is the state-of-the-art method and mostly used in practice.

and two newly developed methods

- *Hybrid optimization method:* The optimization problem (6) is reduced to the manifold of steady states by computing *x*_*s*_(*θ*) numerically.
- *Simulation-based optimization method:* A dynamical system is formulated whose trajectories converge to local optima of the optimization problem (6). The dynamical system is solved using state-of-the-art numerical methods.

To facilitate efficiency and convergence, all methods are provided with the gradients of the objective function,

8 $$\begin{array}{*{20}l} \frac{\partial J}{\partial \theta} &= - \sum\limits_{e=1}^{E} \sum\limits_{j=1}^{N} \sum\limits_{i = 1}^{n_{y}} \frac{\bar{y}_{i}^{e}(t_{j}) - {y_{i}^{e}}\left(t_{j},\theta,{x_{s}^{e}}\right)}{(\sigma_{ij}^e)^{2}} \frac{\partial {y_{i}^{e}}\left(t_{j},\theta,{x_{s}^{e}}\right)}{\partial \theta}\qquad \end{array} $$

9 $$\begin{array}{*{20}l} \frac{\partial J}{\partial {x_{s}^{e}}}&= - \sum\limits_{j=1}^N \sum\limits_{i = 1}^{n_{y}} \frac{\bar{y}_{i}^{e}(t_{j}) - {y_{i}^{e}}\left(t_{j},\theta,{x_{s}^{e}}\right)}{(\sigma_{ij}^e)^{2}} \frac{\partial {y_{i}^{e}}\left(t_{j},\theta,{x_{s}^{e}}\right)}{\partial {x_{s}^{e}}} \end{array} $$

and the gradients of the constraint. The sensitivities of the observable with respect to the parameters and initial conditions, $\frac {\partial y}{\partial \theta }$ and $\frac {\partial y}{\partial {x_{s}^{e}}}$, are computed using the forward sensitivity equations [41].

The optimization problem considered (6) is rather general and allows for the consideration of multiple steady-state constraints, as well as steady-state dose response curves. In the next section the geometry of the steady-state manifold is discussed. Subsequently, the *hybrid optimization method* and the *simulation-based optimization method* are introduced.

### Manifold of steady states

The steady-state constraint defines the steady-state manifold which can be expressed in term of the mapping *x*_*s*_(*θ*,*u*). In the considered setting, the existence of the mapping *x*_*s*_(*θ*,*u*) is ensured but an analytical expression is in general not available. For individual parameters *θ*, the steady state can however be computed by

- solving a feasibility problem (find $x_{s} \in \mathbb {R}^{n_{x}}$ subject to 0=*f*(*x*_*s*_,*θ*,*u*)),
- simulating the dynamical system until the steady state is reached, or
- combining the simulation of the dynamical system with fine-tuning using the Newton-Raphson method [42].

The last two methods are robust and computationally tractable. The computation of the steady state for individual parameters is however not sufficient, as the derivative is also required. To develop a tailored method for solving optimization problems (6), we will exploit the first-order geometry of the manifold of steady states. To this end we consider the sensitivities of the states *x*(*t*) with respect to the parameters *θ*,

10 $$ \begin{aligned} &S(t) = \left(s_{1}(t),s_{2}(t),\ldots, s_{n_{\theta}}(t)\right) \in \mathbb{R}^{n_{x} \times n_{\theta}} \\ & \qquad \text{with} \quad s_{i} := \frac{\partial x}{\partial \theta_{i}} = \left(\frac{\partial x_{1}}{\partial \theta_{i}}, \ldots, \frac{\partial x_{n_{x}}}{\partial \theta_{i}}\right)^{T}. \end{aligned}  $$

in the control conditions. The dynamics of *S* are governed by the forward sensitivity equation

11 $$ \dot{S} = \frac{\partial f}{\partial x} S + \frac{\partial f}{\partial \theta}.  $$

In steady-state, $\dot {S} = 0$, this equation simplifies to

12 $$ S = -\left(\frac{\partial f}{\partial x}\right)^{-1} \frac{\partial f}{\partial \theta},  $$

evaluated at (*θ*,*x*_*s*_(*θ*,*u*),*u*). The invertibility of the Jacobian (*∂f*/*∂x*) follows from local exponential stability of the steady state. This result can also be obtained using the implicit function theorem.

The sensitivity of the steady state with respect to the parameters, *S*, provides a first-order approximation to *x*_*s*_(*θ*,*u*),

13 $$ x_{s}(\theta + r \Delta \theta,u) = x_{s}(\theta,u) + S(\theta,x_{s}(\theta,u),u) r \Delta \theta + O\left(r^{2}\right).  $$

The perturbation direction and the step size are denoted by *Δθ* and *r*, respectively. By reformulating (13) and letting *r*→0, we obtain a dynamical system which evolves on the manifold of steady states,

14 $$ \frac{{dx}_{s}}{dr} = S(\theta,x_{s},u) \Delta \theta.  $$

Given an update direction *Δθ* and a length *r*, (14) provides the steady state for parameter *θ*+*rΔθ* up to the accuracy of the chosen ODE solver. Hence, (14) enables moves on the steady-state manifold, similar to results in [28] for other manifolds.

### Hybrid optimization method

The gold standard for solving optimization problem (6) is to determine an analytical expression for the parameter-dependent steady state. While such an expression is not always available, a straightforward approach is to compute the steady state numerically for given parameters *θ*. This is computationally more demanding than using an analytical expression, but it also yields the reduced optimization problem (7).

**Fig. 2:**
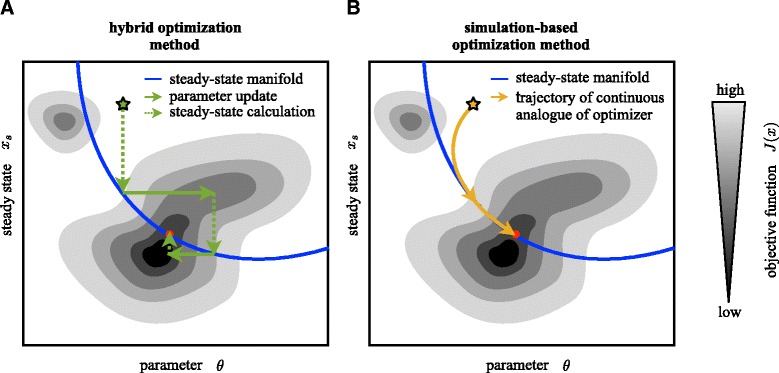
Schematic illustration of **a** the hybrid optimization method and **b** the simulation-based optimization methods. The path of the optimizers are illustrated along with the manifold of steady states and the objective function contour
- Step 2: The steady states $x_{s}^{1,l+1},\ldots, x_{s}^{E,l+1}$ for the parameters *θ*^*l*+1^ are computed using one of the methods discussed in the *Manifold of steady states* section (with starting points $x_{s}^{1,l},\ldots, x_{s}^{E,l}$). This yields the point $\left (\theta ^{l+1},x_{s}^{1,l+1},\ldots,x_{s}^{E,l+1}\right)$ on the steady-state manifold. (Represented as a dotted arrow in Fig. 2a.)
- Step 3: The objective function $J^{l+1} = J\left (\theta ^{l+1},{\newline } x_{s}^{1,l+1},\ldots, x_{s}^{E,l+1}\right)$ is computed for parameters *θ*^*l*+1^ and numerically calculated steady state $x_{s}^{1,l+1},\ldots,x_{s}^{E,l+1}$. This objective function is provided to the local optimizer. (Not represented in Fig. 2a.)

This straightforward approach is visualized in Fig. 2a. As it can be employed in any state-of-the-art optimization method, we denote it as a hybrid optimization method. Starting at a point $(\theta ^{l},{x_{s}^{l}})$, we employ a three-step procedure:

- Step 1: The local optimizer proposes new parameters *θ*^*l*+1^. This yields the point $\left (\theta ^{l+1},x_{s}^{1,l},\ldots,x_{s}^{E,l}\right)$ which is usually not on the manifold of steady states. (Represented as a solid arrow in Fig. 2a.)

The simulation-based retraction to the manifold of steady states (Step 2) reduces the problem dimension and eliminates the constraint. The objective function gradient for this reduced problem is

15 $$ \frac{d J}{d \theta} = \frac{\partial J}{\partial \theta} + \sum_{e=1}^{E} \frac{\partial J}{\partial {x_{s}^{e}}} \frac{\partial {x_{s}^{e}}}{\partial \theta}   $$

with the sensitivity of the steady states with respect to the parameters, $(\partial {x_{s}^{e}}/\partial \theta) = S(\theta,{x_{s}^{e}},{u_{c}^{e}})$, as defined in (12).

The proposed hybrid optimization method possesses all properties and options of the employed local optimizer. In addition, the retraction accuracy *ε*_tol_ of the convergence criteria $||f(x_{s}^{e,l},\theta ^{l},{u_{c}^{e}})||_{2} < \epsilon _{\text {tol}}$ has to be selected.

### Simulation-based optimization method

Instead of using a discrete update as in local optimization, one could also think of choosing a continuous formulation of the update as illustrated in Fig. 2b. The continuous analogue of a gradient descent method is *dθ*/*dr*=−(*dJ*/*dθ*)^*T*^ [30]. This ODE system can be coupled with the dynamical system evolving on the steady-state manifold (14), using *Δθ*=−(*dJ*/*dθ*)^*T*^. More generally we can consider any descent direction *g*(*θ*,*x*_*s*_) in which *J* is decreasing. We obtain the ODE system

16 $$ \begin{aligned} \frac{d\theta}{dr} &= - g(\theta,{x_{s}^{1}},\ldots,{x_{s}^{E}}) \\ \frac{d{x_{s}^{e}}}{dr} &= S(\theta,{x_{s}^{e}},{u_{c}^{e}}) \frac{d\theta}{dr}, \quad e = 1,\ldots,E, \end{aligned}  $$

with the steady-state sensitivity $S\left (\theta,{x_{s}^{e}},{u_{c}^{e}}\right)$. Initialization of (16) in a point $\left (\theta _{0},x_{s,0}^{1},\ldots,x_{s,0}^{E}\right)$ fulfilling (2) yields a trajectory evolving on the steady-state manifold, along which the objective function decreases.

The formulation (16) avoids the repeated simulation-based retractions used by the hybrid optimization method presented in the previous section, however it also bears two disadvantages: (i) An appropriate initial point (*θ*_0_,*x*_*s*,0_) has to be determined by solving (2); and (ii) numerical errors can result in a divergence from the steady-state manifold. To address these problems, we introduce the term *λf*(*θ*,*x*_*s*_) which locally retracts the state of the system to the manifold by exploiting the stability properties of the steady state. This yields the system

17 $$ \begin{aligned} \frac{d\theta}{dr} &= - g\left(\theta,{x_{s}^{1}},\ldots,{x_{s}^{E}}\right) \\ \frac{d{x_{s}^{e}}}{dr} &= \hat{S}\left(\theta,{x_{s}^{e}},{u_{c}^{e}}\right) \frac{d\theta}{dr} + \lambda f\left(\theta,{x_{s}^{e}},{u_{c}^{e}}\right), \,\, e = 1,\ldots,E. \end{aligned}  $$

For this modified system we do not require that the initial point (*θ*_0_,*x*_*s*,0_) fulfill the steady-state constraint (2), hence, the Jacobian $\nabla _{x} f|_{(\theta,x_{s})}\phantom {\dot {i}\!}$ might not be invertible. To address this, we define

18 $$ \hat{S}(\theta,{x_{s}^{e}},{u_{c}^{e}}) = -\left(\frac{\partial f}{\partial x}\right)^{+} \frac{\partial f}{\partial \theta}  $$

in which (*∂f*/*∂x*)^+^ denotes the Moore–Penrose pseudoinverse of (*∂f*/*∂x*) at $(\theta,{x_{s}^{e}},{u_{c}^{e}})$. On the steady-state manifold, the Jacobian is invertible and we recover the standard steady-state sensitivity. For a large retraction factor *λ*≫0, the state $(\theta,{x_{s}^{1}},\ldots,{x_{s}^{E}})$ is retracted quickly to the steady-state manifold.

In this manuscript we consider two possible choices for the descent direction:

- Gradient descent: $g(\theta,x_{s}) = - \frac {dJ}{d\theta }$ and
- Newton-type descent: $g(\theta,x_{s}) = - \left (F + \mu I \right)^{-1} \frac {dJ}{d\theta }$.

The Newton-type descent exploits the Fisher Information Matrix *F* [43]. The Fisher Information matrix is an approximation to the Hessian of the objective function. This approximation can be computed from first-order sensitivities and is positive semi-definite.

For the gradient descent we established local exponential stability of local optima for appropriate choice of *λ* for a broad class of problems (see Additional file 1: Section 1). This implies that the trajectories of the system converge to the local optima of the optimization problem (6). We expect that a similar result can be derived for the Newton-type descent. Though this is not yet available, we included the Newton type descent as we expect – similar to classical optimizers – faster convergence.

For local optimization of (6), the dynamical system (17) has to be simulated for *r*→*∞*. For this, implicit methods with adaptive step-size selection and error control should be employed as (17) might be stiff. Appropriate numerical methods are implemented among others in MATLAB and the SUNDIALS package [44]. These simulations are stopped as soon as the convergence criterion max{∥*dθ*/*dr*∥,∥*dx*_*s*_/*dr*∥}<*ε*_tol_ is met.

### Implementation

The different methods are implemented in MATLAB and provided in an Additional file 2. The local optimization is performed using the MATLAB routine fmincon.m. fmincon is supplied with the objective function value and the values of the constraint, as well as the respective analytical derivatives. The continuous analogue used for simulation-based optimization is simulated using the MATLAB ODE solver ode15s. The computationally intensive simulation of the perturbation experiments and the numerical calculation of the steady state is performed using the SUNDIALS package [44] which was accessed using the MATLAB Toolbox AMICI (https://github.com/AMICI-developer/AMICI). Default settings are used for fmincon.m and the simulation routines unless stated otherwise. The convergence tolerance for the hybrid optimization method is set to *ε*_tol_=10^−9^ in the simulation example and the first application example and to *ε*_tol_=10^−13^ in the second application example. For the simulation-based optimization methods it is set to *ε*_tol_=10^−6^.

### Experimental data

To evaluate the performance of the proposed methods, we use two application examples with experimental data. In the first application example, we consider a dataset for NGF-induced ERK signaling in primary sensory neurons which was published by Andres et al. [45]. In the second application example, we use novel data for Raf/MEK/ERK signaling in HeLa cells after release from S-phase arrest. This novel experimental data for Raf/MEK/ERK signaling in HeLa cells was acquired using the following methods.

**Cell culture.** HeLa cells were obtained from the American Type Culture Collection (Manassas, VA). Cells were maintained in RPMI 1640 supplemented with 10 % fetal bovine serum.

**Cell synchronization at the G1/S border.** HeLa cells were synchronized at the G1/S border using an aphidicolin treatment. In brief, cells grown on Petri dishes were incubated in medium supplemented with aphidicolin (0,3 g/ml; Calbiochem) for 18 h. Afterward, cells were released from the S-phase arrest by washing with serum-free medium and were refed with growth medium.

**Protein extraction of cells.** Whole-cell extracts were obtained by solubilizing cells in hot protein sample buffer (95 °C). After 10 min of incubation at 95 °C, extracts were placed on ice and centrifuged (16,000 *μ*g, 15 min, and 4 °C). Samples were subjected to SDS-PAGE.

**Western blotting.** Equal amounts of proteins were separated by SDS-PAGE and blotted onto nitrocellulose membranes (Pall, Dreieich, Germany). After blocking with 0.5 % blocking reagent (Roche Diagnostics), filters were probed with specific antibodies. Proteins were visualized with IRdye-coupled secondary antibodies using the Li-COR Odyssey system. Protein bands were quantified using ImageJ.

**Antibodies** Commercially available antibodies used in this study were: anti-ERK rabbit polyclonal, anti-phospho-p44/42 MAPK (ERK1/2) (Thr202/Tyr204) rabbit polyclonal, and anti-phospho-MEK1/2 (Ser217/221) rabbit polyclonal (all from Cell Signaling). The tubulin-specific mouse monoclonal antibody was from Millipore. The IRdye-coupled secondary antibodies were from Li-COR Biosciences.

## Results

In the following, we illustrate the behavior of the proposed optimization method. Furthermore, the performance of the proposed methods will be compared to standard constrained and unconstrained optimization methods. For this purpose, we consider a simulation example for which the ground truth is known. Furthermore, we test the methods on two application examples using real data.

### Simulation example: Conversion process

In this section, we illustrate the proposed optimization methods by studying parameter estimation for a conversion process from steady-state data. Conversion processes are among the most common motifs in biological systems, therefore particularly interesting, and provide a simple test case.

We consider the conversion process

19 $$ \mathrm{A} \underset{\theta_{2}}{\overset{\theta_{1} u}{\rightleftarrows}} \mathrm{B},  $$

with parameters $\theta = \left (\theta _{1}, \theta _{2} \right) \in \mathbb {R}_{+}^{2}$ and input $u \in \mathbb {R}_{+}$. Assuming conservation of mass ([A] + [B]=1) and mass action kinetics, the temporal evolution of the concentration of biochemical species *A*, *x*= [A], is governed by

20 $$ \begin{aligned} \frac{dx}{dt} &= \theta_{2} - (\theta_{1} u + \theta_{2}) x\\ y&=x \end{aligned}  $$

with $x_{0} \in \mathbb {R}_{+}$ denoting the initial concentration. The steady state of model (20) is

21 $$ x_{s}(\theta,u) = \frac{\theta_{2}}{\theta_{1} u + \theta_{2}}.   $$

To illustrate the properties of the hybrid and the simulation-based optimization methods, we consider the estimation of the parameters *θ* from artificial time-resolved data for *y*. The artificial data are obtained by simulation of (20) for *θ*=(4,1) and *u*=0.4 at the time points *t*_*j*_= [0,0.1,0.5,1,2], starting from the steady state of the control condition *u*_*c*_=1 at *t*=0. Assuming unit variance for observation errors, yields the optimization problem

22 $$ \begin{aligned} \min_{\theta,x_{s}} &\, J(\theta,x_{s}) := \frac{1}{2} \sum_{j = 1}^{N}\left(\bar{y}(t_{j}) - y(t_{j},\theta,x_{s})\right)^{2}\\ \mathrm{s.t.} \, &\, 0 = \theta_{2} - (\theta_{1} +\theta_{2}) x_{s}, \end{aligned}   $$

in which $\bar {y}(t_{j})$ denotes the measured concentration of A at time point *t*_*j*_ and *y*(*t*_*j*_,*θ*,*x*_*s*_) denotes the solution of (20) for initial conditions *x*(0)=*x*_*s*_ and input *u*=0.4 at time point *t*_*j*_.

#### Illustration of hybrid optimization method

The hybrid optimization method evaluates the steady state numerically but exploits the gradients of the objective functions (15). To this end the objective function gradient, *dJ*/*dθ*,

23 $$ \begin{aligned} \frac{d J}{d \theta} &= - \sum_{j = 1}^{N}\left(\bar{y}(t_{j}) - y(t_{j},\theta,x_{s})\right)\\ &\qquad \left(\frac{\partial y(t_{j},\theta,x_{s})}{\partial \theta} + \frac{\partial y(t_{j},\theta,x_{s})}{\partial x_{s}} \frac{\partial x_{s}}{\partial \theta}\right) \end{aligned}  $$

and the local sensitivities of the steady state for *u*=1,

24 $$ \begin{aligned} S(\theta,x_{s},1) &= \left(\frac{\partial x_{s}}{\partial \theta_{1}}, \frac{\partial x_{s}}{\partial \theta_{2}} \right) \\ &= \left(\frac{- x_{s}}{\theta_{1} + \theta_{2}}, \frac{1 - x_{s}}{\theta_{1} + \theta_{2}} \right) \end{aligned}  $$

are derived. The local sensitivities depend merely on derivatives of the vector field and can be computed without knowledge of an analytical expression of the steady state.

**Fig. 3:**
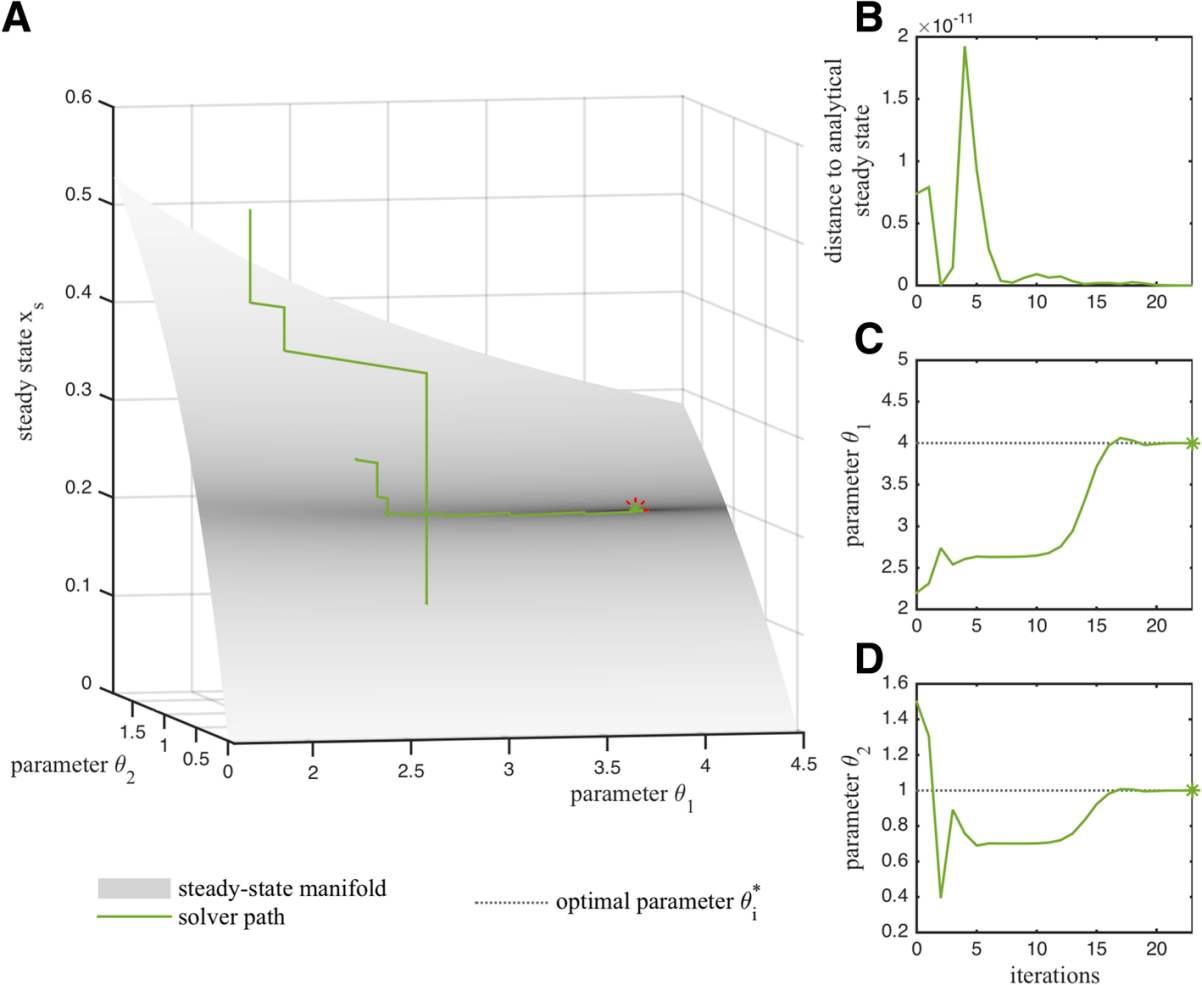
Illustration of the hybrid optimization method for the conversion process (20). **a** Path of the hybrid optimization method (*full lines*), the true optimum (*red star*), and the steady-state manifold (surface, (21)) are shown. The objective function values are indicated by the surface coloring. The optimizer path is partially covered by the steady state manifold. **b** The distance to the analytical steady state as well as **c**, **d** the path of the parameters (*full lines*), their endpoints (*stars*) and optimal parameter value (*dotted lines*) are depicted

The trajectory of the hybrid optimization method is illustrated in Fig. 3. At the end of each iteration, the simulation-based retraction ensures that the parameter-state pair is on the steady-state manifold (Fig. 3a and b). On the steady-state manifold, the optimizer reaches a narrow valley for *θ*_1_ and *θ*_2_ and then moves along the valley to reach the optimum (Fig. 3a, c and d). The behavior is similar for other starting points.

#### Illustration of simulation-based optimization method

For simulation-based optimization the continuous analogue of the gradient descent method is derived. This yields the dynamical system

25 $$ \begin{aligned} \frac{d\theta_{1}}{dr} &= -\sum_{j = 1}^{N}\left(\bar{y}(t_{j}) - y(t_{j},\theta,x_{s})\right)\\ &\qquad \left(\frac{\partial y(t_{j},\theta,x_{s})}{\partial \theta_{1}} + \frac{\partial y(t_{j},\theta,x_{s})}{\partial x_{s}} s_{1}\right) \\ \frac{d\theta_{2}}{dr} &= -\sum_{j = 1}^{N}\left(\bar{y}(t_{j}) - y(t_{j},\theta,x_{s})\right)\\ &\qquad \left(\frac{\partial y(t_{j},\theta,x_{s})}{\partial \theta_{2}} + \frac{\partial y(t_{j},\theta,x_{s})}{\partial x_{s}} s_{2}\right) \\ \frac{{dx}_{s}}{dr} &= \left(\frac{- x_{s}}{\theta_{1} + \theta_{2}}, \frac{1 - x_{s}}{\theta_{1} + \theta_{2}} \right) \left(\begin{array}{c} \frac{d\theta_{1}}{dr}\\ \frac{d\theta_{2}}{dr} \end{array}\right)+\\ &\qquad \lambda (\theta_{2} - (\theta_{1} u + \theta_{2}) x_{s}), \end{aligned}  $$

with initial conditions *θ*_1_(0)=*θ*_1,0_, *θ*_2_(0)=*θ*_2,0_ and *x*_*s*_(0)=*x*_*s*,0_. It can be verified that the objective function *J* is locally strictly convex in *θ* – the parameters are locally identifiable – and that the model (20) is asymptotically stable. Accordingly, system (25) converges to a local optimum of the constrained optimization problem (22) (see Additional file 1: Theorem 1).

**Fig. 4:**
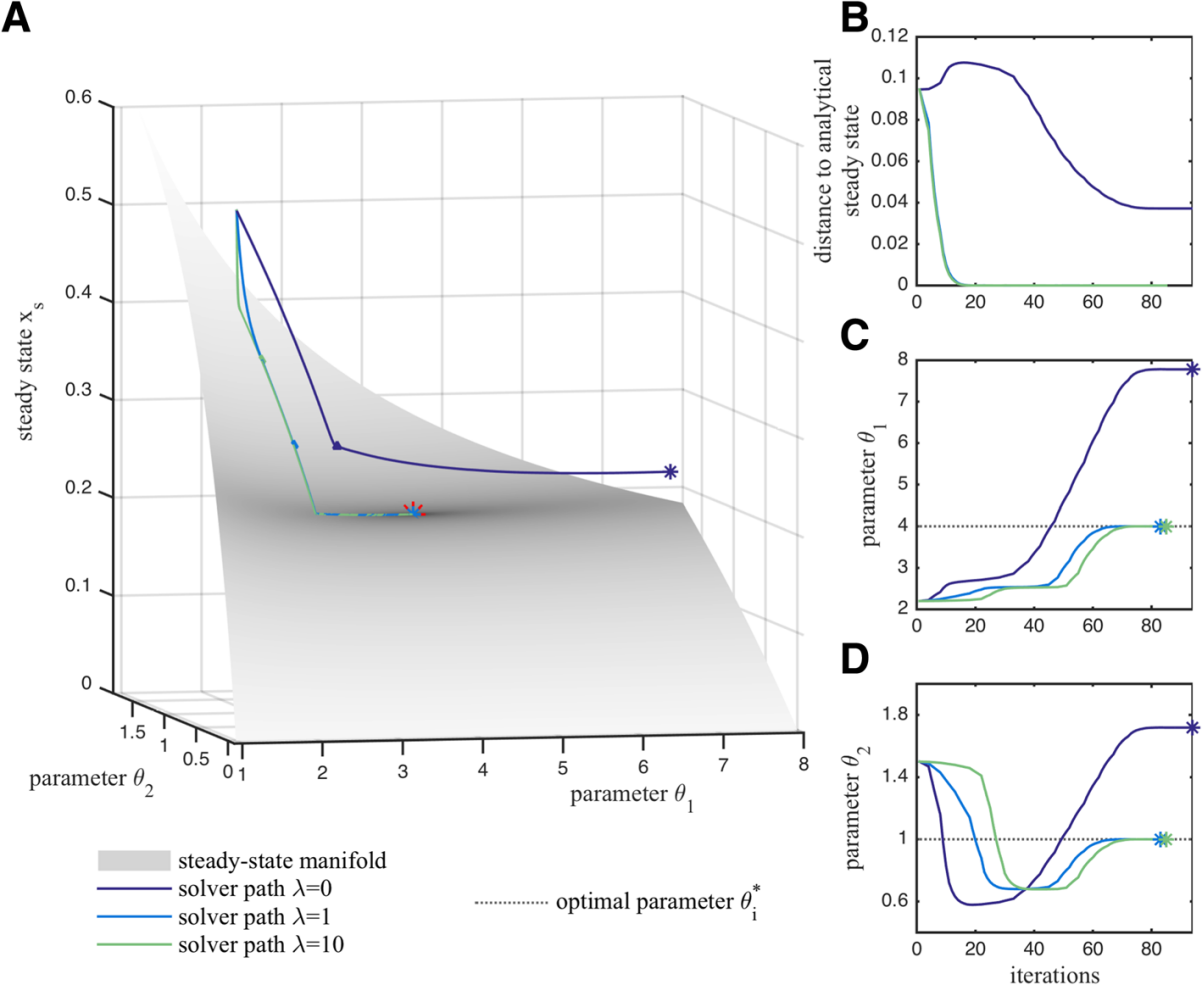
Illustration of the simulation-based optimization method for the conversion process (20). **a** Trajectory of the continuous analogue for different retraction factors *λ* (*full lines*), the endpoints (stars), the true optimum (*red star*), and the steady-state manifold (surface, (21)) are shown. The objective function values are indicated by the surface coloring. **b** The distance to the analytical steady state as well as **c**, **d** the path of the parameters (*full lines*), their endpoints (*stars*) and optimal parameter value (*dotted lines*) are depicted

To illustrate the simulation-based optimization method we simulate the continuous analogue of the gradient descent method. Exemplary trajectories are depicted in Fig. 4. We find that for retraction factors *λ*>0, the states (*θ*_1_,*θ*_2_,*x*_*s*_)^*T*^ converge to the optimal solution. As retraction renders the steady-state manifold (21) attractive, also for initial conditions (*θ*_1,0_,*θ*_2,0_,*x*_*s*,0_)^*T*^ which do not fulfill the steady-state condition, fast convergence to the steady-state manifold can be achieved using *λ*≫1 (Fig. 4a and b). For large retractions (*λ*≫1), the dynamic consists of two phases: (Phase 1) the state *x* converges quickly to the parameter-dependent steady state (21) (Fig. 4a and b); and (Phase 2) the state (*θ*_1_,*θ*_2_,*x*_*s*_)^*T*^ moves along the steady-state manifold to the global optimum (Fig. 4a, c and d).

### Application example 1: NGF-induced ERK signaling in primary sensory neurons

To evaluate and compare existing and proposed local optimization methods for problems with steady-state constraints, we analyze NGF-induced ERK phosphorylation in primary sensory neurons. Primary sensory neurons are among others used to investigate pain sensitization in response to inflammation. During inflammation a cocktail of stimuli is present, including NGF. NGF binds to cellular receptors and induces the ERK phosphorylation [45]. This modulates neuronal activity by triggering ion channel phosphorylation and protein expression [46].

Growth-factor induced ERK signaling is a potential target for novel pain therapies [47] and therefore of high practical relevance. In addition, this application is well-suited for the evaluation of the methods as NGF dose-response curves at late time points have been recorded. These data provide multiple steady-state constraints for the thorough assessment of the methods. In the following, we will compare the performance of unconstrained, constrained, hybrid and simulation-based optimization in the presence of multiple steady-state constraints.

#### Experimental data for NGF-induced ERK phosphorylation

ERK phosphorylation in response to different concentrations of NGF was previously quantified using quantitative automated microscopy [45]. This technique provides single-cell data from which population average data can be derived. These population average data are highly reproducible and quantitative but provide merely the relative ERK phosphorylation in comparison to the control as no calibration curve is employed. The unknown scaling constant is denoted by *s*.

#### Mathematical model of NGF-induced ERK phosphorylation

**Fig. 5:**
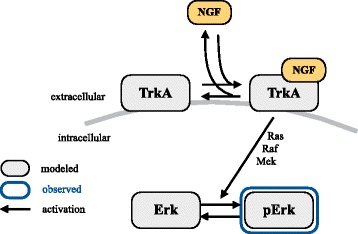
Schematic of the model considered for NGF-induced ERK phosphorylation in primary sensory neurons

NGF induces ERK phosphorylation by binding to the NGF receptor TrkA. The complex TrkA:NGF activates Ras which in turn phosphorylates Raf. pRaf phosphorylates MEK and pMEK phosphorylates ERK (Fig. 5). While all these steps are considered in complex models [48, 49], a previous analysis revealed that the intermediate steps do not have to be modeled to capture the measured data [34]. We therefore use the model introduced in [34],

26 $$ \begin{aligned} \frac{{dx}_{1}}{dt} &= k_{1} u \left(k_{3} [\text{TrkA}]_{0} - x_{1}\right) - k_{2} x_{1}, \\ \frac{{dx}_{2}}{dt} &= \left(x_{1} + k_{4}\right) \left(s [\text{ERK}]_{0} - x_{2}\right) - k_{5} x_{2}, \\ y &= x_{2}, \end{aligned}  $$

to describe the activities of the NGF receptor TrkA (*x*_1_) and ERK phosphorylation (*x*_2_) in response to NGF stimulation, *u*= [NGF]_0_. This model possesses a minimal number of model parameters, *θ*=(*k*_1_,*k*_2_,*k*_3_[TrkA]_0_,*k*_4_,*s*[ERK]_0_,*k*_5_,*σ*^2^), and is structurally identifiable. For details on the model, we refer to the Additional file 1: Section 2 and the original publication [34]. The experimental noise *ε* is assumed to be normally distributed with the unknown variance *σ*^2^, $\epsilon \sim \mathcal {N}(0,\sigma ^{2})$.

The parameter-, and input-dependent steady state of (26) is given by

27 $$ \begin{aligned} x_{s,1}(\theta,u) &= k_{3} [\!\text{TrkA}]_{0} \frac{k_{1} [\text{NGF}]_{0}}{k_{1} [\text{NGF}]_{0} + k_{2}},\\ x_{s,2}(\theta,u) &= s [\!\text{ERK}]_{0} \frac{x_{s,1}(\theta) + k_{4}}{x_{s,1}(\theta,u) + k_{4} + k_{5}}. \end{aligned}  $$

This steady state exists for all positive parameters and is exponentially stable.

#### Parameter estimation problem with multiple steady-state constraints

In this study, the unknown parameters $\theta \in \mathbb {R}_{+}^{7}$ and the states *x*_*s*,1_ and *x*_*s*,2_ for each considered input of NGF are inferred from published dose response data [45] using ML estimation. The dataset contains 6 different NGF doses, yielding an optimization problem with 7+2·6=19 optimization variables and 2·6=12 nonlinear equality constraints. This nonlinear optimization problem is solved using multi-start local optimization. The local optimization is performed using unconstrained, constrained and hybrid optimization as well as simulation-based optimization using gradient and Newton-type descent. Bounds and scales for the parameters are provided in the Additional file 1: Table S1.

**Fig. 6:**
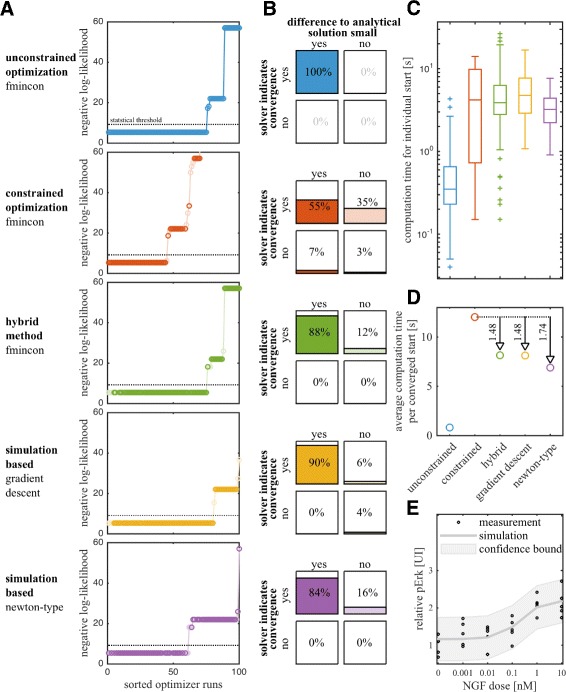
Comparison of optimization methods for NGF-induced ERK activation model. **a** Final objective function values (*darker color* in steady-state, *lighter color* not in steady-state), **b** comparison of convergence criteria with respect to steady-state constraint, **c** computation time for 100 runs and **d** average computation time per converged start of unconstrained optimization method (fmincon), constrained optimization algorithm (fmincon), the hybrid optimization method and the proposed simulation-based optimization methods with gradient descent and newton method based updates and **e** the best fit to the data are depicted

To assess the convergence properties, the constraint satisfaction/violation and the computation time, the local optimization methods were initialized with the same 100 sampled starting points. The results are summarized in Fig. 6. Additionally, we assessed the dependence of the convergence properties on *λ*. The results can be found in the Additional file 1: Section 5.

#### The convergence properties of unconstrained, hybrid and simulation-based optimization are comparable

To assess the convergence of the optimization method, we sort and visualize the objective function values achieved in the individual optimizer starts (Fig. 6a). In addition, we determine the percentage of converged starts. A start is considered to be converged if the final point cannot be rejected compared to the ML estimate using the likelihood ratio test with a significance level of 0.05.

As expected, we find that the gold standard – the unconstrained optimization method – shows the best convergence properties. It converges in 75 % of the starts to the global optimum. A similar convergence is achieved by the proposed methods, hybrid optimization and simulation-based optimization using gradient descent. The third proposed method – simulation-based optimization using Newton-type descent – displays intermediate convergence properties (60 % of the starts converged to the global optimum). The state-of-the-art method – constrained optimization – exhibits the poorest convergence. It converges in 45 % of the starts. Hence, the proposed optimization methods are superior to constrained optimization regarding convergence to the global optimum.

Beyond differences in the convergence to the global optimum, the convergence to local optima differs. The results of unconstrained, constrained and hybrid optimization reveal three local optima. The local optima with the worst objective function values are hardly found using simulation-based optimization, indicating altered regions of attraction.

#### Hybrid and simulation-based optimization provide reliable estimates of the steady states

The individual optimization methods enforce the steady-state constraints differently. What all methods have in common is that the steady-state constraint *f*(*x*_*s*_,*θ*,*u*_*s*_)=0 is relaxed to a constraint on the norm of the vector field, i.e., ||*f*(*x*_*s*_,*θ*,*u*_*s*_)||_2_<*ε*_*f*_. Accordingly, parameter-state pairs returned by the optimization methods usually do not fulfill steady-state constraints exactly. Different optimization methods might even achieve different accuracies. In addition, a bound for the difference of the estimated steady state *x*_*s*_ for a parameter *θ* and the true steady state *x*_*s*_(*θ*), *Δx*_*s*_=*x*_*s*_−*x*_*s*_(*θ*), is usually not available.

We studied the relation of the solver indicating convergence based on the vector field (||*f*||_2_<*ε*_*f*_) and the difference of the estimated to the analytical steady-state being small (||*Δx*_*s*_||_2_<*ε*_*x*_) for the different optimization methods. In our opinion a good optimizer should achieve equivalence of the two criteria. This would mean that enforcing the constraint of the vector field ensures a good approximation of the steady state. The result is depicted in Fig. 6b for a tolerance of 10^−6^.

The unconstrained optimization uses an analytical expression of the steady state and therefore the two criteria are identical. Hybrid and simulation-based optimization also achieved a good agreement of both criteria, with ∼85 %. In ∼15 % of the cases, the solver indicates convergence based on the vector field constraint but the steady-state estimate is off (||*Δx*_*s*_||_2_>*ε*_*x*_). The precise percentage depends heavily on the retraction factor *λ* for the simulation-based optimization method. For the constrained optimization, all possible combinations are observed and the two criteria agree in merely 55 % of the runs. In summary, the results indicate that the proposed methods provide reliable estimates for the steady states while constrained optimization yields many inconsistent parameter-state pairs.

#### Hybrid and simulation-based optimization are faster than constrained optimization

A key performance metric for local optimizers is the average computation time per converged start. This metric summarizes convergence properties (Fig. 6a and b) and computation times for individual starts (Fig. 6c). It is computed by dividing the overall computation time for the multi-start optimization by the number of converged starts. This measure of optimizer performance is depicted in Fig. 6d.

Unconstrained optimization using a (usually not available) analytical expressions for steady states is most efficient. The individual runs are fast and the percentage of converged starts is high. Hybrid and simulation-based methods are roughly 10 times slower but these methods can be applied if analytical expressions for steady states are not available. Furthermore, these methods are 1.5 times faster than constrained optimization due to the improved convergence rate. Additionally, the fit to the data for the optimal parameters is convincing (Fig. 6e). Accordingly, we conclude that hybrid and simulation-based optimization are promising approaches in the presence of multiple steady-state constraints.

### Application 2: Raf/MEK/ERK signaling in HeLa cells after release from S-phase arrest

In this section, we study Raf/MEK/ERK signaling in HeLa cells after release from S-phase arrest. Experimental studies revealed that cell-cycle is, among others, controlled by Raf/MEK/ERK signaling [50, 51]. The signaling dynamics in different cell-cycle phases as well as the cell-cycle-dependent relevance of feedback mechanisms [52] are however still not completely unraveled although a more thorough understanding could provide valuable insights into treatment resistance [52]. Using the new data and model selection we study the relevance of negative feedback from phospho-ERK to Raf activation during G1/S phase transition.

In addition to its biological relevance, the Raf/MEK/ ERK pathway is well-suited for the evaluation of the proposed optimization methods and the comparison to state-of-the-art methods. The pathway is nonlinear, yielding a nonlinear and non-convex optimization problem. Furthermore, we will consider a synchronized cell population which reached a steady state before the start of the experiment. Accordingly, a steady-state constraint has to be enforced and fitted along with time-resolved data for perturbation experiments.

#### Experimental data for Raf/MEK/ERK signaling after release from S-phase arrest

To study the Raf/MEK/ERK pathway, HeLa cells were synchronized at the G1/S border using an aphidicolin treatment. After synchronization was achieved, aphidicolin was removed and the dynamics of phospho-MEK and phospho-ERK were quantified using Western blotting. This was repeated after treatment with Sorafenib and UO126 to explore the dynamic range of the pathway. Sorafenib is an inhibitor of Raf kinases [53] and UO126 is a highly selective inhibitor of MEK [54].

As Western blots are merely semi-quantitive, they provide the relative activity of phospho-MEK and phospho-ERK at different time points and under different conditions. The unknown scaling constants differ between blots and measured species. For a detailed discussion of characteristics of Western blot data we refer to [55].

#### Mathematical model for Raf/MEK/ERK signaling after release from S-phase arrest

**Fig. 7:**
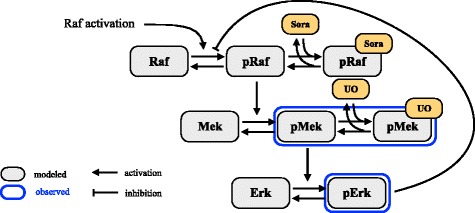
Schematic of the model considered for the Raf/MEK/ERK signaling after release from S-phase arrest

Raf/MEK/ERK signaling is induced by myriads of intra- and extracellular signals [51, 56]. These signals converge on the level of Raf kinase, which they phosphorylate. The phosphorylated Raf kinase phosphorylates MEK, which in turn phosphorylates ERK. ERK induces downstream signaling and can down-regulate the Raf activity [49]. The latter establishes a negative feedback loop [52, 57]. The activity of Raf and MEK can be inhibited by Sorafenib and UO126, respectively. The pathway is illustrated in Fig. 7.

In this section we develop a model for Raf/MEK/ERK signaling which accounts for the core proteins as well as their inhibition with Sorafenib and UO126. The model considers the six reactions:

28 $$ \begin{aligned} &\mathrm{R}_{1}: &&\text{Raf} \rightarrow \text{pRaf}, && \text{rate} = k_{1,\max}(t) \xi(t) [\!\text{Raf}], \\ &\mathrm{R}_{2}: &&\text{pRaf} \rightarrow \text{Raf}, && \text{rate} = k_{2} [\!\text{pRaf}], \\ &\mathrm{R}_{3}: &&\text{MEK} \rightarrow \text{pMEK}, && \text{rate} = \frac{k_{3} K_{2} [\!\text{pRaf}]}{K_{2} + [\!\text{sora}]} [\!\text{MEK}], \\ &\mathrm{R}_{4}: &&\text{pMEK} \rightarrow \text{MEK}, && \text{rate} = k_{4} [\!\text{pMEK}], \\ &\mathrm{R}_{5}: &&\text{ERK} \rightarrow \text{pERK}, && \text{rate} = \frac{k_{5} K_{3} [\!\text{pMEK}] }{K_{3} + [\!\text{UO126}]} [\!\text{ERK}], \\ &\mathrm{R}_{6}: &&\text{pERK} \rightarrow \text{ERK}, && \text{rate} =k_{6} [\!\text{pERK}]. \end{aligned}  $$

The upstream signaling is summarized in the time-dependent rate constant *k*_1,max_(*t*) with the flexible parameterization

29 $$ k_{1,\max}(t) = k_{1,0} + k_{1,1}\left(1-e^{-\frac{t}{\tau_{1}}}\right)e^{-\frac{t}{\tau_{2}}}  $$

(as proposed in the Data2Dynamics toolbox [58]). The effects of Sorafenib and UO126 are captured by a reduction in the kinase activity of pRaf and pMEK (R_4_ and R_6_).

Experimental studies proved an inhibition of Raf phosphorylation by pERK [52]. This feedback is however context-dependent [49]. To study the importance of this feedback during the G1/S phase transition, we considered two model hypotheses:

- Inhibition of Raf phosphorylation by pERK: $\xi (t) = \frac {K_{1}}{K_{1} + [\text {pERK}]}$
- No inhibition: *ξ*(*t*)=1

Using mass conservation and reformulations explained in detail in Additional file 1: Section 3 we arrive at the ODE model

30 $$ \begin{aligned} \frac{{dx}_{1}}{dt} &= k_{1,\max}(t) \xi(t) (1 - x_{1}) - k_{2} x_{1} \\ \frac{{dx}_{2}}{dt} &= \frac{k_{3} [\!\text{Raf}]_{0} K_{2} x_{1}}{K_{2} + [\!\text{sora}]} (1 - x_{2}) - k_{4} x_{2}\\ \frac{{dx}_{3}}{dt} &= \frac{k_{5} [\!\text{MEK}]_{0} K_{3} x_{2}}{K_{3} + [\text{UO126}]} (1 - x_{3}) - k_{6} x_{3} \\ y_{1,b} &= s_{1,b} [\!\text{MEK}]_{0} x_{2}\\ y_{2,b} &= s_{2,b} [\!\text{ERK}]_{0} x_{3} \end{aligned}  $$

for the relative phosphorylation levels *x*_1_= [pRaf]/[Raf]_0_, *x*_2_= [pMEK]/[MEK]_0_ and *x*_3_= [pERK]/[ERK]_0_ and the input *u*=([sora],[UO126]). The model for the relative phosphorylation levels does not depend explicitly on the total abundances [Raf]_0_, [MEK]_0_ and [ERK]_0_ but only on products and ratios of these parameters with other parameters, e.g., *k*_3_[Raf]_0_. Defining these products and ratios as new parameters eliminates non-identifiabilities and reduces the number of parameters. Each Western blot, indexed by *b*=1,…,4, provides time-resolved data for *y*_1,*b*_ and *y*_2,*b*_ for a combination of different experimental conditions. The measurement noise is assumed to be normally distributed and its variance is estimated from the experimental data. As all parameters are non-negative, a log-parameterization is used for parameter estimation [15]. The states of the reformulated model are between 0 and 1. Details regarding parameters and initial conditions are provided in the Additional file 1: Table S2.

In addition to the kinetic, scaling and noise parameters, the initial conditions of the models for H1 and H2 are unknown. As the cells are however arrested in S-phase with *k*_1,max_(0)=*k*_1,0_ and *u*=0, the initial conditions are the corresponding steady states. After significant manual preprocessing of the steady-state constraints, analytical expressions *x*_*s*_(*θ*) for the steady-states as a function of the other parameters could be calculated with symbolic math toolboxes. These analytical expressions are provided in the Additional file 1: Equation (10), (11).

#### Parameter estimation problem with multiple perturbation datasets

**Fig. 8:**
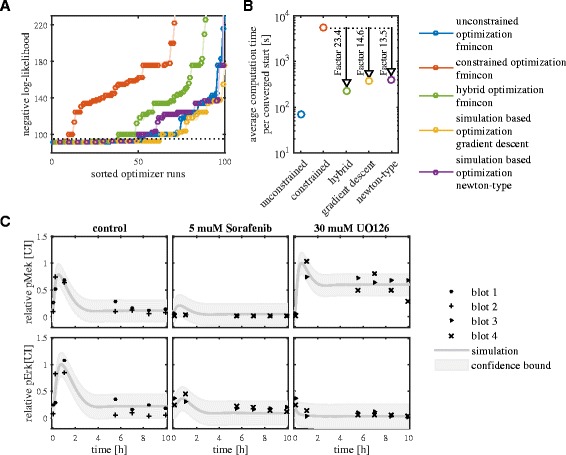
Parameter estimation results for Raf/MEK/ERK signaling in HeLa cells. **a** Convergence and **b** computational efficiency of local optimization methods for the model with the negative feedback loop (H1). **c** Best fit of the model with the negative feedback loop (H1) to data for three different treatment conditions. pMEK and pERK signals are rescaled with the respective maximum activity and the light gray area indicates 2- *σ* interval of the measurement noise

We inferred the model parameters and initial conditions from the Western blot data using ML estimation. The dataset provides time-resolved data for three conditions (control & two perturbations), all starting from the same steady-state. The optimization problem is solved using multi-start local optimization. The local optimization was performed using unconstrained, constrained and hybrid optimization as well as simulation-based optimization using gradient and Newton-type descent each method starting at the same points. The starting points for local optimizations were obtained using Latin hypercube sampling (see Additional file 1: Table S2). The maximal number of iterations and function evaluations performed by fmincon were increased to 2000 and 2000*n*_*θ*_ for the unconstrained and constrained optimization. For the hybrid optimization, the maximal number of iterations was increased to 2000. The results for 100 starts of the local optimizations for the model of H1 are depicted in Fig. 8a and b.

#### Hybrid and simulation-based optimization outperforms constrained optimization

Unconstrained optimization using the analytical expression for the steady state – the gold standard – converged in ∼50 % of the starts (Fig. 8a). Hybrid and simulation-based optimization methods achieved a percentage of converged starts comparable to the gold standard (40–60 %), but without requiring an analytical expression for the steady state. Constrained optimization – the state-of-the-art – converged in less than 10 % of the starts, resulting in a relatively large computation time per converged start (Fig. 8b). Even though hybrid and simulation-based optimization were slower than the gold standard, they were more than 10 times faster than constrained optimization. Hence, the proposed optimization methods also outperform constrained optimization for this problem.

A detailed comparison of the proposed methods revealed that simulation-based optimization using gradient descent achieved the highest percentage of converged starts. Hybrid optimization required however fewer simulations of the perturbation experiments – the time-consuming step – rendering this method computationally more efficient. Simulation-based optimization using Newton-type descent was the least efficient of the proposed methods. This might be related to the challenges in tuning the regularization parameters.

#### Model selection reveals importance of negative feedback

The model with negative feedback (H1) fits the experimental data (Fig. 8c). It captures the transient phosphorylation of MEK and ERK after release from S-phase arrest, the reduced ERK phosphorylation in the presence of Sorafenib and UO126. Furthermore, the increased MEK phosphorylation after UO126 treatment is explained via a decrease in the strength of the negative feedback which is caused by the reduced abundance of pERK. The model without the negative feedback loop (H2) is not able to capture the difference between the control condition and the simulation with UO126. The value of the Bayesian Information Criterion (BIC) [59] is 278.4 for the model with negative feedback (H1) and 317.4 for the model without negative feedback (H2). The difference of 39.0 indicates a strong preference for H1 [60]. The same conclusion is reached using the Akaike Information Criterion (AIC) [61]. We conclude that Raf phosphorylation is inhibited by pERK during G1/S phase transition.

To conclude, in this section we illustrated the proposed hybrid and simulation-based optimization methods. The applicability of the methods was demonstrated by studying relevant biological problems. The comparison with state-of-the-art methods revealed convergence and computational efficiency. The study of Raf/MEK/ERK signaling using the methods underlined the feedback regulation of ERK phosphorylation during cell cycle progression.

## Discussion

Optimization problems with steady state constraints arise in many biology applications for a wide range of models. For some models an analytical expression for the steady state can be derived and used to eliminate the steady-state constraints [18]. While this is favorable, it is not always possible. In cases in which no analytical expressions are available, the vector of optimization variables contains the unknown parameters as well as the corresponding steady states. The optimizers have to evolve on the non-linear manifold, the set of steady states. In this manuscript, we propose a hybrid optimization method and a continuous analogue to solve optimization problems with steady-state constraints more efficiently. This simulation-based method exploits the local geometry of the steady-state manifold for optimization.

The proposed hybrid and simulation-based optimization methods are evaluated using three models for biological processes. Following a simple illustration example, an application with multiple steady-state constraints and an application with time-resolved data for multiple perturbation conditions are considered. For this rich set of scenarios we find that the hybrid and simulation-based optimization methods possess improved convergence properties in comparison to standard constrained optimization methods implemented in the MATLAB routine fmincon. We expect that the proposed methods also outperform alternative optimization routines (e.g. IPOPT [62]), this, however, remains to be analyzed. The proposed optimization methods yield convergence properties comparable to those of unconstrained optimization methods exploiting an analytical expression for the steady state. However, if analytical expressions for the steady state can be determined using available methods [18, 25, 26], unconstrained optimization should be used as the computation time is lower. The proposed methods are also applicable to a broader class of problems for which no analytical expression for the steady state is available. Furthermore, the method directly allows for multiple steady-state constraints. Unlike methods based on sequential geometric programming [63, 64], steady-state and kinetic data can be handled.

Beyond the evaluation of the proposed methods, our experimental and computational analysis of novel data for Raf/MEK/ERK signaling after release for S-phase arrest provided new insights. Parameter estimation and model comparison indicate that the negative feedback from ERK to Raf phosphorylation is also active at the G1/S border. This complements previous knowledge of stimulus-, context-dependence of this stimulus [49] and its relevance for the robustness of MAPK signaling in tumor cells [52].

The implementation of the hybrid optimization method employed in this study is a simulation-based retraction operator. Alternatively, efficient and accurate schemes combining simulation and local optimization could be employed to compute steady states and sensitivities [42]. This should improve the computational efficiency further. To relax the stability assumption for the steady state, conservation relations can be incorporated in the local optimization scheme.

For the simulation-based optimization method we established local asymptotic stability of optimal points using perturbation theory. This result is however restricted to the gradient-type descent and locally convex objective functions. The latter implies local practical identifiability. The theoretical properties of the Newton-type descent and the properties in the presence of practical and structural non-identifiability remain to be analyzed. Preliminary results and the applications suggest that in the presence of non-identifiabilities the simulation-based optimization method always yields a point on the non-identifiable subspace. Furthermore, the available proof shows the retraction factor *λ* has to be chosen large enough to ensure convergence. However, as too large *λ* will result in a stiff system, an intelligent choice of *λ* is necessary.

The simulation example and the applications possess a unique exponentially stable steady state. However, preliminary results suggest that the methods also achieve good convergence for dynamical systems with multiple stable steady states and bifurcations [65] (see Additional file 1: Section 6). The theoretical analysis of the proposed methods and a detailed performance evaluation for dynamical systems with such properties remains to be addressed.

Beyond parameter estimation, the proposed optimization methods can also accelerate practical indentifiability analysis and uncertainty quantification by speeding up optimization runs in bootstrapping uncertainty analysis [66, 67] and profile likelihood calculation [68]. In addition, Bayesian uncertainty analysis using Markov chain Monte Carlo sampling [11] can profit from an efficient initial optimization prior to the sampling. This has been shown to reduce the warm-up and to improve convergence [69]. For a more detailed discussion of identifiabilty and uncertainty analysis methods, we refer to recent comparative studies [70, 71].

## Conclusion

In summary, the proposed optimization methods are promising alternatives to constrained optimization for optimization problems with steady-state constraints. They are applicable to a wide range of ODE-constrained optimization problems [34, 72] and can – unlike methods which rely on an analytical expression for the steady state – be extended to PDE constrained optimization problems [73]. The availability of the MATLAB code will facilitate the application and extension of the methods, as well as the integration in toolboxes such as Data2Dynamics [58] and COPASI [74]. Accordingly, our study has a strong potential influence on the analysis of optimization problems with steady-state constraints in practice.

## Abbreviations

Not applicable.

## Additional files

Additional file 1 Supporting Information S1. This document provides a detailed description of the stability proof and the derivation of the different pathway models. Furthermore, the parameter and bounds are listed, the influence of the retraction factor on convergence and run time as well as the behavior of the proposed methods in the presence of multiple steady states and bifurcations is illustrated. (PDF 577 kb)

Additional file 2 Code S1. This zip-file contains the MATLAB code used for the simulation example (conversion process) and the application examples (NGF-induced Erk signaling in primary sensory neurons and Raf/MEK/ERK signaling in HeLa cells after release from S-phase arrest) presented in the paper. We provide implementations for the hybrid optimization and simulation-based optimization methods, the models and the optimization. In addition to the implementation, also all data and result files (.mat,.csv) are included. (ZIP 25965 kb)
