# Supplementary material for: Tailored parameter optimization methods for ordinary differential equation models with steady-state constraints
Source: BMC Syst Biol. 2016 Aug 22;10(1):80. doi: 10.1186/s12918-016-0319-7 (PMC4994295; doi:10.1186/s12918-016-0319-7)
Supplement: Additional file 1 — Supporting Information S1. This document provides a detailed description of the stability proof and the derivation of the different pathway models. Furthermore, the parameter and bounds are listed, the influence of the retraction factor on convergence and run time as well as the behavior of the proposed methods in the presence of multiple steady states and bifurcations is illustrated. (PDF 577 kb) [file 12918_2016_319_MOESM1_ESM.pdf]

Supplement to

Tailored parameter optimization methods for ordinary differential equation models with steady-state constraints

Anna Fiedler[1,2], Sebastian Raeth[3], Fabian J. Theis[1,2], Angelika Hausser[3] and Jan Hasenauer[1,2]

[1]Helmholtz Zentrum München - German Research Center for Environmental Health, Institute of Computational Biology, 85764 Neuherberg, Germany

[2]Technische Universität München, Center for Mathematics, Chair of Mathematical Modeling of Biological Systems, 85748 Garching, Germany

[3] University of Stuttgart, Stuttgart Research Center Systems Biology (SRCSB), 70569 Stuttgart, Germany

1 Analysis of simulation-based optimization method

In the following, we assess the local stability and convergence of simulation-based optimization with gradient descent to a local optimum $(\theta^*, x_s(\theta^*))$ for a single steady-state constraint corresponding to an arbitrary but fixed input u_c. For easier notation we drop the dependence of x_s, f and J on u_c in the notation. The theorem can be extended to several steady states. We consider the following four conditions.

Assumption 1.1. *There exists a locally isolated root $x_s(\theta)$ of the equation $f(\theta, x_s(\theta)) = 0$ for all $(\theta - \theta^*) \in B_c = \{\tilde{\theta} \in \mathbb{R}^{n_\theta} | \|\tilde{\theta}\| \leq c\} \subseteq \mathbb{R}^{n_\theta}$.*

This root is a steady state of the ODE model.

Assumption 1.2. *The steady state $x_s(\theta)$ is locally exponentially stable uniformly in θ, with $(\theta - \theta^*) \in B_c$.*

Accordingly, $\exists \zeta_0, \gamma, k > 0$ such that $\|x(t) - x_s(\theta)\| \leq k\|x(0) - x_s(\theta)\| \exp(-\gamma t)$, $\forall (x(0) - x_s(\theta)) \in B_{\zeta_0} := \{\tilde{\Delta}_s(0) \in \mathbb{R}^{n_x} | \|\tilde{\Delta}_s(0)\| < \zeta_0\}$ and $\forall (\theta - \theta^*) \in B_c$. This implies that $\exists \zeta \geq \zeta_0$ such that $(x(t) - x_s(\theta)) \in B_\zeta := \{\tilde{\Delta}_s(t) \in \mathbb{R}^{n_x} | \|\tilde{\Delta}_s(t)\| < \zeta\}$ (Khalil, 2002).

Assumption 1.3. *There exists a neighborhood around θ^*, $(\theta - \theta^*) \in B_c$, in which the objective function evaluated on the steady-state manifold $x_s(\theta)$, $J(\theta, x_s(\theta))$, is locally strictly convex in θ.*

This implies that the parameters θ are locally structurally identifiable and that solutions to $d\theta/dr = -dJ/d\theta|_{(\theta, x_s(\theta))}$ converge to the optimal point θ^* for initial points $(\theta(0) - \theta^*) \in B_c$.

Assumption 1.4. *The functions $(dJ/d\theta)|_{(\theta, x_s)}$, $f(\theta, x_s)$ and $x_s(\theta)$ and their partial derivatives up to order 2 are bounded for $(\theta - \theta^*) \in B_c$ and $(x_s - x_s(\theta)) \in B_\zeta$.*

Using these assumptions we find:

Theorem 1.1. *Let Assumptions 1.1 - 1.4 be satisfied. Then there exists a λ^* such that for all $\lambda > \lambda^*$ a local minimum (θ^*, x_s^*) of the optimization problem is a locally exponentially stable steady state of the simulation-based optimization system.*

Proof. To prove Theorem 1.1 we use perturbation theory (Khalil, 2002). We define $\varepsilon = \lambda^{-1}$ and shift the optimum to the origin using the linear state transformation,

$$\tilde{\theta} = \theta - \theta^* \quad \text{and} \quad \tilde{x}_s = x_s - x_s(\theta^*).$$

This yields the singular perturbed system

$$\begin{aligned}\frac{d\tilde{\theta}}{dr} &= -\frac{dJ}{d\tilde{\theta}}^T =: \tilde{F}(\tilde{\theta}, \tilde{x}_s, \varepsilon)\\ \varepsilon\frac{d\tilde{x}_s}{dr} &= -\varepsilon\hat{S}\frac{dJ}{d\tilde{\theta}}^T + f =: \tilde{G}(\tilde{\theta}, \tilde{x}_s, \varepsilon),\end{aligned} \tag{1}$$

with $dJ/d\tilde{\theta}$, f and $\hat{S}$ evaluated at $(\tilde{\theta} + \theta^*, \tilde{x}_s + x_s(\theta^*))$. The system (1) captures the dynamics of the deviances from the optimal parameter, $\tilde{\theta}$, and the deviances from the steady state for the optimal parameter, $\tilde{x}_s$. Furthermore, it possesses the following properties:

(i) $\tilde{F}(0, 0, \varepsilon) = 0$ and $\tilde{G}(0, 0, \varepsilon) = 0$ as the objective function gradient vanishes, $0 = (dJ/d\tilde{\theta})|^T_{(\theta^*, x_s(\theta^*))}$ and as the steady-state condition is fulfilled, $0 = f(\theta^*, x_s(\theta^*))$. Both follows from optimality of θ^* and Assumption 1.1.

(ii) The equation $0 = \tilde{G}(\tilde{\theta}, \tilde{x}_s, 0)$ has the isolated root $\tilde{x}_s(\tilde{\theta}) = x_s(\tilde{\theta} + \theta^*) - x_s(\theta^*)$ (Assumption 1.1) with $\tilde{x}_s(0) = 0$.

(iii) The functions $\tilde{F}$, $\tilde{G}$, and x_s and their partial derivatives up to order 2 are bounded for $(\tilde{x}_s - x_s(\tilde{\theta})) \in B_\zeta$ (Assumption 1.4).

(iv) The origin of the reduced systems

$$\frac{d\tilde{\theta}}{dr} = F(\tilde{\theta}, \tilde{x}_s(\tilde{\theta}), \varepsilon) = -\left.\frac{dJ}{d\tilde{\theta}}\right|^T_{(\tilde{\theta}+\theta^*, x_s(\tilde{\theta})+x_s(\theta^*))}$$

obtained for $\varepsilon = 0$ is locally exponentially stable, with $x_s(\tilde{\theta}) + x_s(\theta^*) = x_s(\tilde{\theta} + \theta^*)$ from (ii). This follows from strict local convexity of the objective at θ^* (Assumption 1.3).

(v) The boundary-layer system is derived from (1) using the transformation $\tilde{\Delta}_s = \tilde{x}_s - \tilde{x}_s(\tilde{\theta}) (= \tilde{x}_s - \tilde{x}_s(\tilde{\theta}))$, yielding

$$\begin{aligned}\frac{d\tilde{\theta}}{dr} &= -\frac{dJ}{d\tilde{\theta}}^T\\ \varepsilon\frac{d\tilde{\Delta}_s}{dr} &= \varepsilon(S - \hat{S})\frac{dJ}{d\tilde{\theta}}^T + f,\end{aligned}$$

with $dJ/d\tilde{\theta}$, f and $\hat{S}$ evaluated at $(\tilde{\theta}+\theta^*, \tilde{\Delta}_s + x_s(\tilde{\theta}+\theta^*))$ and S evaluated at $(\tilde{\theta}+\theta^*, x_s(\tilde{\theta}+\theta^*))$. After rescaling of the simulation time, $\rho = r/\varepsilon$, and setting $\varepsilon = 0$, we obtain the boundary-layer system

$$\frac{d\tilde{\Delta}_s}{d\rho} = f(\tilde{\theta} + \theta^*, \tilde{\Delta}_s + x_s(\tilde{\theta} + \theta^*)).$$

The origin of this boundary-layer system is locally exponentially stable, uniformly in $\tilde{\theta}$ as the steady state $x_s(\theta)$ of $\frac{dx}{dt} = f(x, \theta, u)$ is exponentially stable uniformly in $\theta = \tilde{\theta} + \theta^*$ (Assumption 1.2).

The properties (i)-(v) are the prerequisites of (Khalil, 2002, Theorem 9.3), establishing the existence of $\varepsilon^* > 0$ such that for all $\varepsilon < \varepsilon^*$ systems of type (1) are locally exponentially stable. As stability properties are conserved under the performed transformations, we obtain for $\varepsilon = \lambda^{-1}$ the Theorem 1.1. □

Stability and convergence are not affected by the approximation of the steady-state sensitivity via $\hat{S}$.

2 Derivation of the mathematical model for NGF-induced ERK signaling

We consider in the following a model which accounts for five reactions,

$$\begin{aligned}
&R_1: && \text{TrkA} + \text{NGF} \rightarrow \text{TrkA:NGF}, && \text{rate} = k_1[\text{TrkA}][\text{NGF}],\\
&R_2: && \text{TrkA:NGF} \rightarrow \text{TrkA} + \text{NGF}, && \text{rate} = k_2[\text{TrkA:NGF}],\\
&R_3: && \text{ERK} \rightarrow \text{pERK}, && \text{rate} = k_3[\text{TrkA:NGF}][\text{ERK}],\\
&R_4: && \text{ERK} \rightarrow \text{pERK}, && \text{rate} = k_4[\text{ERK}],\\
&R_5: && \text{pERK} \rightarrow \text{ERK}, && \text{rate} = k_5[\text{pERK}].
\end{aligned} \tag{2}$$

These reactions describe binding of NGF to TrkA (R_1 and R_2), TrkA:NGF-mediated ERK phosphorylation (R_3), basal ERK phosphorylation (R_4) and ERK dephosphorylation (R_5). Brackets indicate the concentration of a biochemical species. The input is the initial NGF concentration, $u = [\text{NGF}]_0$, and the measured output is the relative pERK concentration,

$$y = s[\text{pERK}]. \tag{3}$$

The experimental noise ϵ is assumed to be normally distributed with the unknown variance σ^2, $\epsilon \sim \mathcal{N}(0, \sigma^2)$.

In accordance with previous publications, we assume conservation of mass ($[\text{NGF}] + [\text{TrkA:NGF}] = [\text{NGF}]_0$, $[\text{TrkA}] + [\text{TrkA:NGF}] = [\text{TrkA}]_0$ and $[\text{ERK}] + [\text{pERK}] = [\text{ERK}]_0$) and excess of NGF ($[\text{NGF}]_0 \gg [\text{TrkA}]_0$) (see (Hasenauer *et al.*, 2014) and references therein). Under these assumptions the activities of the NGF receptor TrkA and ERK are captured by

$$\begin{aligned}
\frac{dx_1}{dt} &= k_1 u \left(k_3[\text{TrkA}]_0 - x_1\right) - k_2 x_1, \\
\frac{dx_2}{dt} &= \left(x_1 + k_4\right)\left(s[\text{ERK}]_0 - x_2\right) - k_5 x_2, \\
y &= x_2,
\end{aligned} \tag{4}$$

in which $x_1 = k_3[\text{TrkA:NGF}]$ and $x_2 = s[\text{pERK}]$. This model possesses a minimal number of parameters, $\theta = (k_1, k_2, k_3[\text{TrkA}]_0, k_4, s[\text{ERK}]_0, k_5, \sigma^2)$ and is structurally identifiable. For details on the model, we refer to the original publication (Hasenauer *et al.*, 2014).

3 Derivation of the mathematical model for Raf/MEK/ERK signaling

We consider a model for Raf/MEK/ERK signaling which accounts for the core proteins and considers the six reactions:

$$\begin{aligned}
&R_1: && \text{Raf} \to \text{pRaf}, && \text{rate} = k_{1,\max}(t)\xi(t)[\text{Raf}], \\
&R_2: && \text{pRaf} \to \text{Raf}, && \text{rate} = k_2[\text{pRaf}], \\
&R_3: && \text{MEK} \to \text{pMEK}, && \text{rate} = \frac{k_3 K_2 [\text{pRaf}]}{K_2 + [\text{sora}]}[\text{MEK}], \\
&R_4: && \text{pMEK} \to \text{MEK}, && \text{rate} = k_4[\text{pMEK}], \\
&R_5: && \text{ERK} \to \text{pERK}, && \text{rate} = \frac{k_5 K_3 [\text{pMEK}]}{K_3 + [\text{UO126}]}[\text{ERK}], \\
&R_6: && \text{pERK} \to \text{ERK}, && \text{rate} = k_6[\text{pERK}].
\end{aligned} \tag{5}$$

The upstream signaling is summarized in the time-dependent rate constant $k_{1,\max}(t)$ for which the flexible parameterization

$$k_{1,\max}(t) = k_{1,0} + k_{1,1}\left(1 - e^{-\frac{t}{\tau_1}}\right) e^{-\frac{t}{\tau_2}}. \tag{6}$$

(as proposed in the Data2Dynamics toolbox (Raue *et al.*, 2015)) is used. The effects of sorafenib and UO126 are captured by a reduction in the kinase activity of pRaf and pMEK (R_4 and R_6).

Experimental studies proved an inhibition of Raf phosphorylation by pERK (Fritsche-Guenther *et al.*, 2011). This feedback is however context-dependent (Santos *et al.*, 2007). To study the importance of this feedback during the G1/S phase transition, we considered two model hypotheses:

H1 Inhibition of Raf phosphorylation by pERK: $\xi(t) = \frac{K_1}{K_1 + [\text{pERK}]}$

H2 No inhibition: $\xi(t) = 1$

We assume that Raf, MEK and ERK are conserved ($[\text{Raf}] + [\text{pRaf}] = [\text{Raf}]_0$, $[\text{MEK}] + [\text{pMEK}] = [\text{MEK}]_0$ and $[\text{ERK}] + [\text{pERK}] = [\text{ERK}]_0$), yielding the ODE model

$$\begin{aligned}
\frac{d[\text{pRaf}]}{dt} &= k_{1,\max}(t)\xi(t)([\text{Raf}]_0 - [\text{pRaf}]) - k_2[\text{pRaf}] \\
\frac{d[\text{pMEK}]}{dt} &= \frac{k_3 K_2 [\text{pRaf}]}{K_2 + [\text{sora}]}([\text{MEK}]_0 - [\text{pMEK}]) - k_4[\text{pMEK}] \\
\frac{d[\text{pERK}]}{dt} &= \frac{k_5 K_3 [\text{pMEK}]}{K_3 + [\text{UO126}]}([\text{ERK}]_0 - [\text{pERK}]) - k_6[\text{pERK}],
\end{aligned} \tag{7}$$

with experimental input $u = ([\text{sora}], [\text{UO126}])$. The four collected Western blots, $b = 1, \ldots, 4$, provide time-resolved relative data for different experimental inputs,

$$\begin{aligned} y_{1,b} &= s_{1,b}[\text{pMEK}] \\ y_{2,b} &= s_{2,b}[\text{pERK}] \end{aligned} \tag{8}$$

with unknown, blot-dependent scaling constants $s_{1,b}$ and $s_{2,b}$. The experimental noise for $y_{1,b}$ and $y_{2,b}$ is assumed to be normally distributed and proportional to the scaling constant, $\epsilon_{1,b} \sim \mathcal{N}(0, s_{1,b}^2\sigma_1^2)$ and $\epsilon_2 \sim \mathcal{N}(0, s_{2,b}^2\sigma_2^2)$. The unknown variances are denoted by σ_1^2 and σ_2^2.

for H1 and H2, model (7)-(8) possesses more than 20 parameters. Several of these parameters are structurally non-identifiable, including the absolute abundances of Raf, MEK and ERK. To circumvent these non-identifiablilities, we reformulate the model in terms of the fractions of phosphorylated proteins: $x_1 = [\text{pRaf}]/[\text{Raf}]_0$, $x_2 = [\text{pMEK}]/[\text{MEK}]_0$ and $x_3 = [\text{pERK}]/[\text{ERK}]_0$. This yields

$$\begin{aligned} \frac{dx_1}{dt} &= k_{1,\max}(t)\xi(t)(1 - x_1) - k_2x_1 \\ \frac{dx_2}{dt} &= \frac{k_3[\text{Raf}]_0K_2x_1}{K_2 + [\text{sora}]}(1 - x_2) - k_4x_2 \\ \frac{dx_3}{dt} &= \frac{k_5[\text{MEK}]_0K_3x_2}{K_3 + [\text{UO126}]}(1 - x_3) - k_6x_3 \\ y_{1,b} &= s_{1,b}[\text{MEK}]_0x_2 \\ y_{2,b} &= s_{2,b}[\text{ERK}]_0x_3 \end{aligned} \tag{9}$$

with blot index $b = 1, \ldots, 4$ and rescaled experimental noise $\tilde{\epsilon}_{1,b} \sim \mathcal{N}(0, s_{1,b}^2\sigma_1^2/[\text{MEK}]_0^2)$ and $\tilde{\epsilon}_{2,b} \sim \mathcal{N}(0, s_{2,b}^2\sigma_2^2/[\text{ERK}]_0^2)$. The reformulated model does not depend explicitly on the total abundances $[\text{Raf}]_0$, $[\text{MEK}]_0$ and $[\text{ERK}]_0$ but only on products and ratios of these parameters with other parameters, e.g., $k_3[\text{Raf}]_0$. Defining these products and ratios as new parameters eliminates non-identifiabilities and reduces the number of parameters. As all parameter are non-negative, a log-parameterization is used for parameter estimation (Raue *et al.*, 2013). The states of the reformulated model are between 0 and 1.

In addition to the kinetic, scaling and noise parameters, the initial conditions of the models for H1 and H2 are unknown. However, as the cells are arrested in S-phase with $k_{1,\max}(0) = k_{1,0}$ and $u = 0$, the initial conditions are the corresponding steady states. After significant manual preprocessing of the steady-state constraints, analytical expressions $x_s(\theta)$ for the steady states as a function of the other parameters could be calculated with symbolic math toolboxes. For simplicity we define $\tilde{K}_1 = K_1/[\text{ERK}]_0$, $\tilde{k}_3 = k_3[\text{Raf}]_0$ and

$\tilde{k}_5 = k_5[\text{MEK}]_0$. With this the steady state for H1 is given by

$$
\begin{aligned}
x_{s,1}(\theta) = &\Bigg(\tilde{K}_1 k_{1,0} + \Bigg(\tilde{K}_1^2 k_{1,0}^2 + \frac{2\tilde{K}_1^2 k_6 k_{1,0}^2}{\tilde{k}_5} + \frac{\tilde{K}_1^2 k_6^2 k_{1,0}^2}{\tilde{k}_5^2} + \frac{\tilde{K}_1^2 k_4^2 k_6^2 \left(k_{1,0} + k_2\right)^2}{(\tilde{k}_3 \tilde{k}_5)^2} + \\
&\frac{2\tilde{K}_1^2 k_4 k_6^2 k_{1,0} \left(k_{1,0} + k_2\right)}{\tilde{k}_3 \tilde{k}_5^2} + \frac{2\tilde{K}_1^2 k_4 k_6 k_{1,0} \left(k_{1,0} + k_2\right)}{\tilde{k}_3 \tilde{k}_5} + \frac{4\tilde{K}_1 k_2 k_4 k_6 k_{1,0}}{\tilde{k}_3 \tilde{k}_5}\Bigg)^{\frac{1}{2}} + \frac{\tilde{K}_1 k_6 k_{1,0}}{\tilde{k}_5} - \\
&\frac{\tilde{K}_1 k_4 k_6 \left(k_{1,0} + k_2\right)}{\tilde{k}_3 \tilde{k}_5}\Bigg) \Bigg/ \left(2\left(k_2 + \tilde{K}_1 k_{1,0} + \tilde{K}_1 k_2 + \frac{\tilde{K}_1 k_2 k_6}{\tilde{k}_5} + \frac{\tilde{K}_1 k_6 k_{1,0}}{\tilde{k}_5}\right)\right)
\end{aligned}
$$

$$
\begin{aligned}
x_{s,2}(\theta) = &\Bigg(\Bigg(\tilde{K}_1^2 k_{1,0}^2 + \frac{2\tilde{K}_1^2 k_6 k_{1,0}^2}{\tilde{k}_5} + \frac{\tilde{K}_1^2 k_6^2 k_{1,0}^2}{\tilde{k}_5^2} + \frac{\tilde{K}_1^2 k_4^2 k_6^2 \left(k_{1,0} + k_2\right)^2}{(\tilde{k}_3 \tilde{k}_5)^2} + \\
&\frac{2\tilde{K}_1^2 k_4 k_6^2 k_{1,0} \left(k_{1,0} + k_2\right)}{\tilde{k}_3 \tilde{k}_5^2} + \frac{2\tilde{K}_1^2 k_4 k_6 k_{1,0} \left(k_{1,0} + k_2\right)}{\tilde{k}_3 \tilde{k}_5} + \frac{4\tilde{K}_1 k_2 k_4 k_6 k_{1,0}}{\tilde{k}_3 \tilde{k}_5}\Bigg)^{\frac{1}{2}} + \\
&\tilde{K}_1 k_{1,0} + \frac{\tilde{K}_1 k_6 k_{1,0}}{\tilde{k}_5} - \frac{\tilde{K}_1 k_2 k_4 k_6}{\tilde{k}_3 \tilde{k}_5} - \frac{\tilde{K}_1 k_4 k_6 k_{1,0}}{\tilde{k}_3 \tilde{k}_5}\Bigg) \Bigg/ \\
&\Bigg(\Bigg(\tilde{K}_1^2 k_{1,0}^2 + \frac{2\tilde{K}_1^2 k_6 k_{1,0}^2}{\tilde{k}_5} + \frac{\tilde{K}_1^2 k_6^2 k_{1,0}^2}{\tilde{k}_5^2} + \frac{\tilde{K}_1^2 k_4^2 k_6^2 \left(k_{1,0} + k_2\right)^2}{(\tilde{k}_3 \tilde{k}_5)^2} + \frac{2\tilde{K}_1^2 k_4 k_6^2 k_{1,0} \left(k_{1,0} + k_2\right)}{\tilde{k}_3 \tilde{k}_5^2} + \\
&\frac{2\tilde{K}_1^2 k_4 k_6 k_{1,0} \left(k_{1,0} + k_2\right)}{\tilde{k}_3 \tilde{k}_5} + \frac{4\tilde{K}_1 k_2 k_4 k_6 k_{1,0}}{\tilde{k}_3 \tilde{k}_5}\Bigg)^{\frac{1}{2}} + \\
&\tilde{K}_1 k_{1,0} + \frac{\tilde{K}_1 k_6 k_{1,0}}{\tilde{k}_5} + \frac{k_2 k_4}{1\tilde{k}_3}\left(2\tilde{K}_1 + \frac{\tilde{K}_1 k_6}{\tilde{k}_5} + 2\right) + \frac{\tilde{K}_1 k_4 k_{1,0}}{\tilde{k}_3}\left(\frac{k_6}{\tilde{k}_5} + 2\right)\Bigg)
\end{aligned} \tag{10}
$$

$$
\begin{aligned}
x_{s,3}(\theta) = &\Bigg(\Bigg(\tilde{K}_1^2 \left(k_{1,0}\right)^2 + \frac{2\tilde{K}_1^2 k_6 k_{1,0}^2}{\tilde{k}_5} + \frac{\tilde{K}_1^2 k_6^2 k_{1,0}^2}{\tilde{k}_5^2} + \frac{\tilde{K}_1^2 k_4^2 k_6^2 \left(k_{1,0} + k_2\right)^2}{(\tilde{k}_3 \tilde{k}_5)^2} + \\
&\frac{2\tilde{K}_1^2 k_4 k_6^2 k_{1,0} \left(k_{1,0} + k_2\right)}{\tilde{k}_3 \tilde{k}_5^2} + \frac{2\tilde{K}_1^2 k_4 k_6 k_{1,0} \left(k_{1,0} + k_2\right)}{\tilde{k}_3 \tilde{k}_5} \\
&+ \frac{4\tilde{K}_1 k_2 k_4 k_6 k_{1,0}}{\tilde{k}_3 \tilde{k}_5}\Bigg)^{\frac{1}{2}} + \tilde{K}_1 k_{1,0} + \frac{\tilde{K}_1 k_6 k_{1,0}}{\tilde{k}_5} - \frac{\tilde{K}_1 k_2 k_4 k_6}{1\tilde{k}_3 \tilde{k}_5} \\
&- \frac{\tilde{K}_1 k_4 k_6 k_{1,0}}{\tilde{k}_3 \tilde{k}_5}\Bigg) \Bigg/ \Bigg(\left(\frac{k_6}{\tilde{k}_5} + 1\right)\Bigg(\tilde{K}_1^2 k_{1,0}^2 + \frac{2\tilde{K}_1^2 k_6 k_{1,0}^2}{\tilde{k}_5} + \frac{\tilde{K}_1^2 k_6^2 k_{1,0}^2}{\tilde{k}_5^2} + \\
&\frac{\tilde{K}_1^2 k_4^2 k_6^2 \left(k_{1,0} + k_2\right)^2}{(\tilde{k}_3 \tilde{k}_5)^2} + \frac{2\tilde{K}_1^2 k_4 k_6^2 k_{1,0} \left(k_{1,0} + k_2\right)}{\tilde{k}_3 \tilde{k}_5^2} + \\
&\frac{2\tilde{K}_1^2 k_4 k_6 k_{1,0} \left(k_{1,0} + k_2\right)}{\tilde{k}_3 \tilde{k}_5} + \frac{4\tilde{K}_1 k_2 k_4 k_6 k_{1,0}}{\tilde{k}_3 \tilde{k}_5}\Bigg)^{\frac{1}{2}} + \tilde{K}_1 k_{1,0}\left(\frac{k_6}{\tilde{k}_5} + 1\right)^2 \\
&+ \frac{k_2 k_4 k_6}{\tilde{k}_3 \tilde{k}_5}\left(\tilde{K}_1 + \frac{\tilde{K}_1 k_6}{\tilde{k}_5} + 2\right) + \frac{\tilde{K}_1 k_6 k_6 k_{1,0}}{\tilde{k}_3 \tilde{k}_5}\left(\frac{k_6}{\tilde{k}_5} + 1\right)\Bigg).
\end{aligned}
$$

The steady state for H2 is given by

$$x_{s,1}(\theta) = \frac{k_{1,0}}{k_{1,0} + k_2}$$

$$x_{s,2}(\theta) = \frac{\tilde{k}_3 \frac{k_{1,0}}{k_{1,0}+k_2}}{\tilde{k}_3 \frac{k_{1,0}}{k_{1,0}+k_2} + k_4} \tag{11}$$

$$x_{s,3}(\theta) = \left(\tilde{k}_5 \frac{\tilde{k}_3 \frac{k_{1,0}}{k_{1,0}+k_2}}{\tilde{k}_3 \frac{k_{1,0}}{k_{1,0}+k_2} + k_4} \right) / \left(\tilde{k}_5 \frac{\tilde{k}_3 \frac{k_{1,0}}{k_{1,0}+k_2}}{\tilde{k}_3 \frac{k_{1,0}}{k_{1,0}+k_2} + k_4} + k_6 \right).$$

4 Parameters for Application example 1 and Application example 2

The properties of the parameters and initial conditions for the model of NGF-induced ERK phosphorylation are provided in Table 1 and the properties of the parameters for the model of Raf/MEK/ERK signaling after release from S-phase arrest are provided in Table 2.

Supplement Table 1: Lower bounds, upper bounds and scale used for fitting of the parameters of model for NGF-induced ERK phosphorylation.

	Parameter name	Lower bound	Upper bound	Scale used for optimization	Bound used for optimization	Bound used for sampling
Kinetic parameters	k_1	10^{-5}	$10^{3.5}$	$\log_{10}$	yes	yes
	k_2	10^{-5}	$10^{3.5}$	$\log_{10}$	yes	yes
	$k_3[\text{TrkA}]_0$	10^{-5}	$10^{3.5}$	$\log_{10}$	yes	yes
	k_4	10^{-5}	$10^{3.5}$	$\log_{10}$	yes	yes
	$s[\text{ERK}]_0$	10^{-5}	$10^{3.5}$	$\log_{10}$	yes	yes
	k_5	10^{-5}	$10^{3.5}$	$\log_{10}$	yes	yes
Initial conditions	$x_{s,1}$	0	3	lin	no	yes
	$x_{s,2}$	0	3	lin	no	yes

5 Retraction factor

As an example, we studied the influence of the retraction factor λ on the convergence and computation time for Application example 1: NGF-induced ERK phosphorylation. To this end we considered retraction factors varying over several orders of magnitude and performed for each of these λ a multi-start optimization with 100 starts using simulation-based optimization with gradient descent and newton-type descent directions. We used the same setup as in Application example 1. The results are illustrated in Figure 1. In the case of gradient descent, the computation time decreases with increasing λ, which also results in decreasing average computation time per converged start. Choosing $\lambda > 2000$ cannot decrease the average computation time further. It can also be observed that there seems to be an upper limit on the maximal computation time. This is caused by a restriction on the maximal possible function evaluations allowed. In the case of a newton-type descent, increasing λ also decreases the computation time, however, not as drastically as when using gradient descent. Again, also the average computation time per converged start decreases, but plateaus at much smaller values of λ than in the gradient descent case.

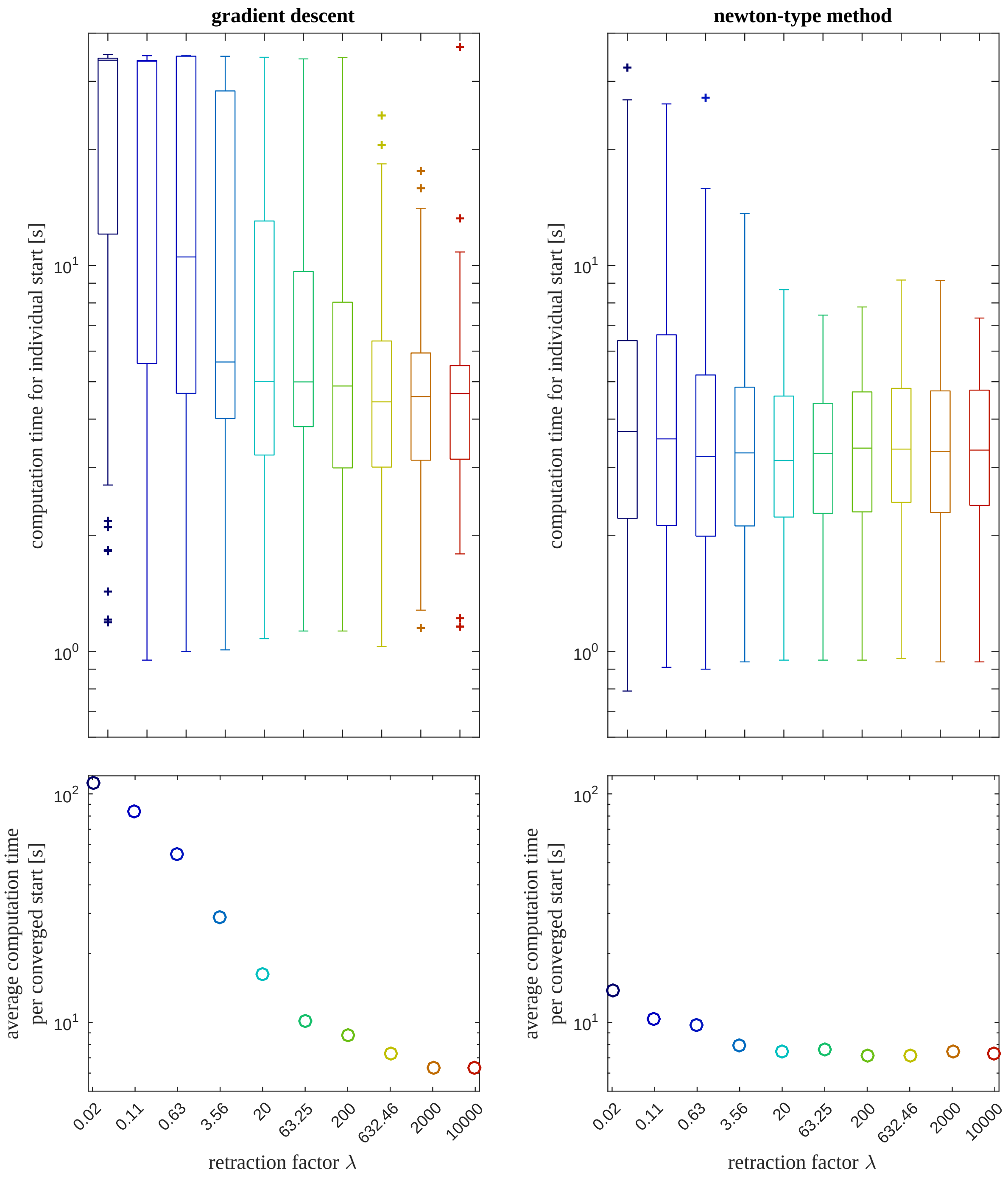

Supplement Figure 1: Comparison of the influence of retraction factor λ on the computation time and the average computation time per converged start for the simulation based optimization method using gradient descent and newton-type descent directions for Application example 1.

Supplement Table 2: Lower bounds, upper bounds and scale used for fitting of the parameters of model for Raf/MEK/ERK signaling.

	Parameter name	Lower bound	Upper bound	Scale used for optimization	Bound used for optimization	Bound used for sampling
Kinetic parameters	$k_{1,1}/k_{1,0}$	10^{-4}	10^{2}	log	yes	yes
	τ_1	10^{-4}	10^{2}	log	yes	yes
	τ_2	10^{-4}	10^{2}	log	yes	yes
	$K_1/[\mathrm{ERK}]_0$	10^{-4}	10^{2}	log	yes	yes
	$k_{1,1}$	10^{-4}	10^{2}	log	yes	yes
	k_2	10^{-4}	10^{2}	log	yes	yes
	K_2	10^{-4}	10^{2}	log	yes	yes
	$k_3[\mathrm{Raf}]_0$	10^{-4}	10^{2}	log	yes	yes
	k_4	10^{-4}	10^{2}	log	yes	yes
	K_3	10^{-4}	10^{2}	log	yes	yes
	$k_5[\mathrm{MEK}]_0$	10^{-4}	10^{2}	log	yes	yes
	k_6	10^{-4}	10^{2}	log	yes	yes
Scaling parameters	$s_{1,b}[\mathrm{MEK}]_0$	10^{-4}	10^{2}	log	yes	yes
	$s_{2,b}[\mathrm{ERK}]_0$	10^{-4}	10^{2}	log	yes	yes
Noise parameters	σ_1^2	10^{-10}	10^{2}	log	yes	yes
	σ_2^2	10^{-10}	10^{2}	log	yes	yes
Initial conditions	$x_{s,1}$	0	1	lin	no	yes
	$x_{s,2}$	0	1	lin	no	yes
	$x_{s,3}$	0	1	lin	no	yes

6 Behavior in the presence of bistability, bifurcations and oscilations

Biological systems often possess multiple stable steady states and non-trivial ω-limit sets, e.g. stable limit cycles. To evaluate the simulation-based optimization method and the hybrid optimization method, we consider a bistable system (with hysteresis) and a system with a Hopf bifurcation.

6.1 Bistable system

To study the performance of the proposed methods in the presence of bistability we considered the system

$$\dot{x} = \frac{x^{20}}{1 + x^{20}} - x + \theta, \qquad x(0) = x_0. \tag{12}$$

as described in (Müller & Kuttler, 2015, Chapter 5, p. 532). We assume direct observation of the state and the objective function $J(x_s, \theta) = (\bar{x}_s - x_s)^2$ with $\bar{x}_s \approx 1.5$. The measurement $\bar{x}_s$ is the larger of the two steady states for $\theta = 0.5$. The resulting minimization problem is

$$\begin{aligned} &\min_{x_s,\theta} J(x_s, \theta) = (\bar{x}_s - x_s)^2 \\ &\text{s.t. } 0 = \frac{x_s^{20}}{1 + x_s^{20}} - x_s + \theta. \end{aligned} \tag{13}$$

The optimization was performed using the hybrid and the simulation-based optimization method.

Figure 2 illustrates the convergence and 5 example trajectories. For both methods we observe good convergence to the optimal point, illustrating good properties in the presence of bistability.

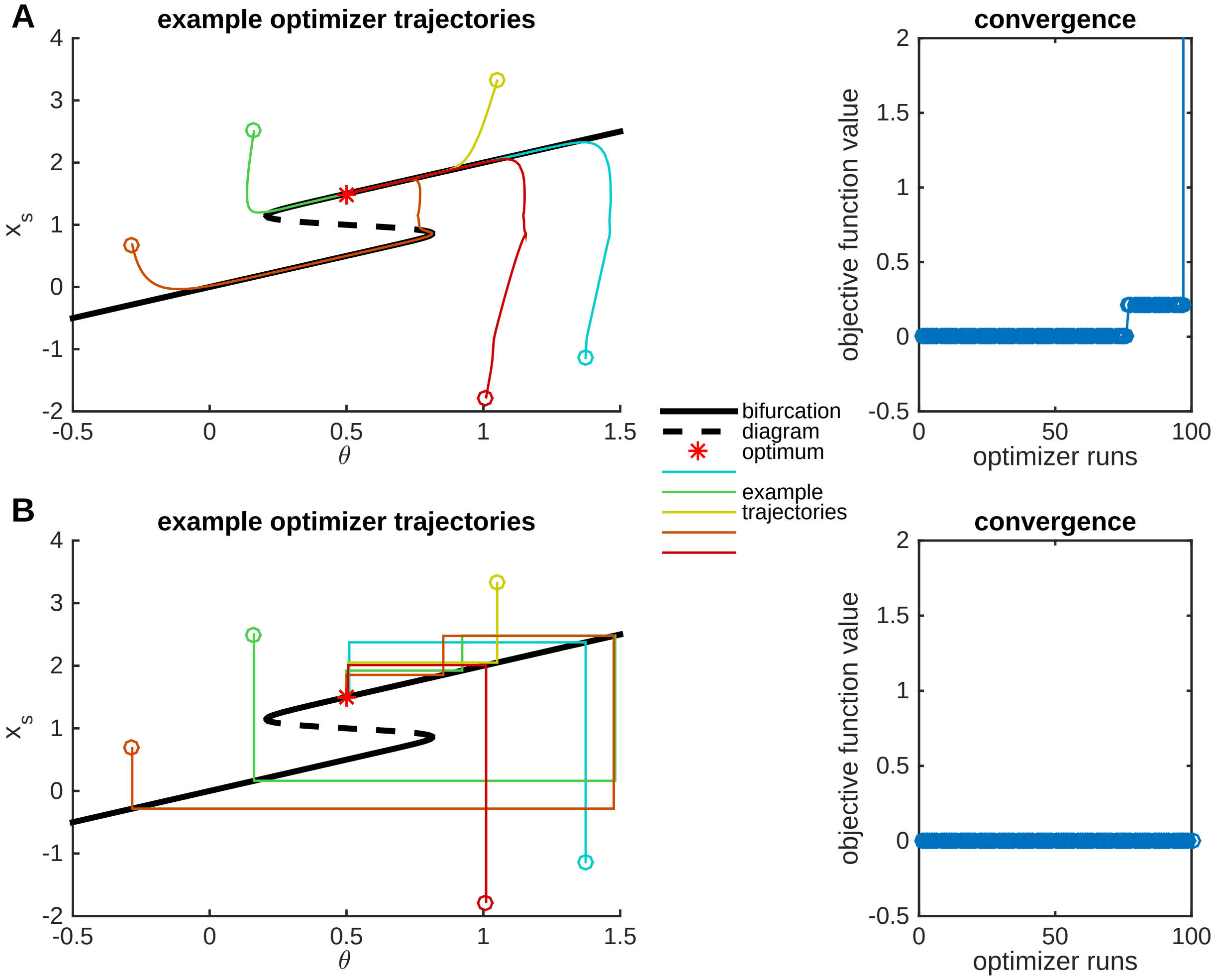

Supplement Figure 2: Evaluation of (A) the simulation-based optimization method and (B) the hybrid optimization method for bistable system. (left) 5 example optimizer trajectories and (right) results of 100 local optimization runs.

6.2 System with Hopf bifurcation

To study the performance of the proposed method in the presence of stable oscillations, we consider the system

$$\dot{x}_1 = -x_2 + x_1(\theta - x_1^2 - x_2^2), \qquad x_1(0) = x_{1,0} \tag{14}$$

$$\dot{x}_2 = x_1 + x_2(\theta - x_1^2 - x_2^2), \qquad x_2(0) = x_{2,0}. \tag{15}$$

This is the normal form for systems with Hopf bifurcation as described in (Müller & Kuttler, 2015, Chapter 2, p. 227). The measurement was taken to be $x_s = (0,0)^T$ which corresponds to the steady state for $\theta \leq 0$. For $\theta > 0$ this system exhibits stable limit cycles and no stable steady state. We considered a least squares objective function yielding the optimization problem

$$\begin{aligned} \min_{x_s,\theta} J(x_s,\theta) &= \sum_{i=1}^{2} x_{s,i}^2 \\ \text{s.t. } 0 &= x_{s,2} + x_{s,1}(\theta - x_{s,1}^2 - x_{s,2}^2) \\ 0 &= x_{s,1} + x_{s,2}(\theta - x_{s,1}^2 - x_{s,2}^2). \end{aligned} \tag{16}$$

The optimization was performed using only the simulation-based optimization method as the simulation in the hybrid optimization step is not converging for $\theta > 0$ as no stable steady state exists. Figure 3 illustrates example optimizer trajectories. The results illustrate a good convergence of the simulation-based optimization method to the set of parameters θ with steady state $x_s = (0,0)^T$, independent of the starting point.

In summary, the analysis of these simple systems indicates that in particular the simulation-based optimization method also facilitates the analysis of models with multiple stable steady states and limit cycles.

References

Fritsche-Guenther, R., Witzel, F., Sieber, A., Herr, R., Schmidt, N., Braun, S., Brummer, T., Sers, C., & Blüthgen, N. (2011). Strong negative feedback from Erk to Raf confers robustness to MAPK signalling. *Mol. Syst. Biol.*, *7*(489).

Hasenauer, J., Hasenauer, C., Hucho, T., & Theis, F. J. (2014). ODE constrained mixture modelling: A method for unraveling subpopulation structures and dynamics. *PLoS Comput. Biol.*, *10*(7), e1003686.

Khalil, H. K. (2002). *Nonlinear Systems*. Upper Saddle River, New Jersey: Prentice Hall, 3rd ed.

Müller, J. & Kuttler, C. (2015). *Methods and Models in Mathematical Biology*. Springer-Verlag Berlin Heidelberg, 1 ed.

Raue, A., Schilling, M., Bachmann, J., Matteson, A., Schelke, M., Kaschek, D., Hug, S., Kreutz, C., Harms, B. D., Theis, F. J., Klingmüller, U., & Timmer, J. (2013). Lessons learned from quantitative dynamical modeling in systems biology. *PLoS ONE*, *8*(9), e74335.

Raue, A., Steiert, B., Schelker, M., Kreutz, C., Maiwald, T., Hass, H., Vanlier, J., Tönsing, C., Adlung, L., Engesser, R., Mader, W., Heinemann, T., Hasenauer, J., Schilling, M., Höfer, T., Klipp, E., Theis, F. J., Klingmüller, U., Schöberl, B., & J.Timmer (2015). Data2Dynamics: a modeling environment tailored to parameter estimation in dynamical systems. *Bioinformatics*.

Santos, S. D. M., Verveer, P. J., & Bastiaens, P. I. H. (2007). Growth factor-induced MAPK network topology shapes Erk response determining PC-12 cell fate. *Nat. Cell Biol.*, *9*(3), 324–330.

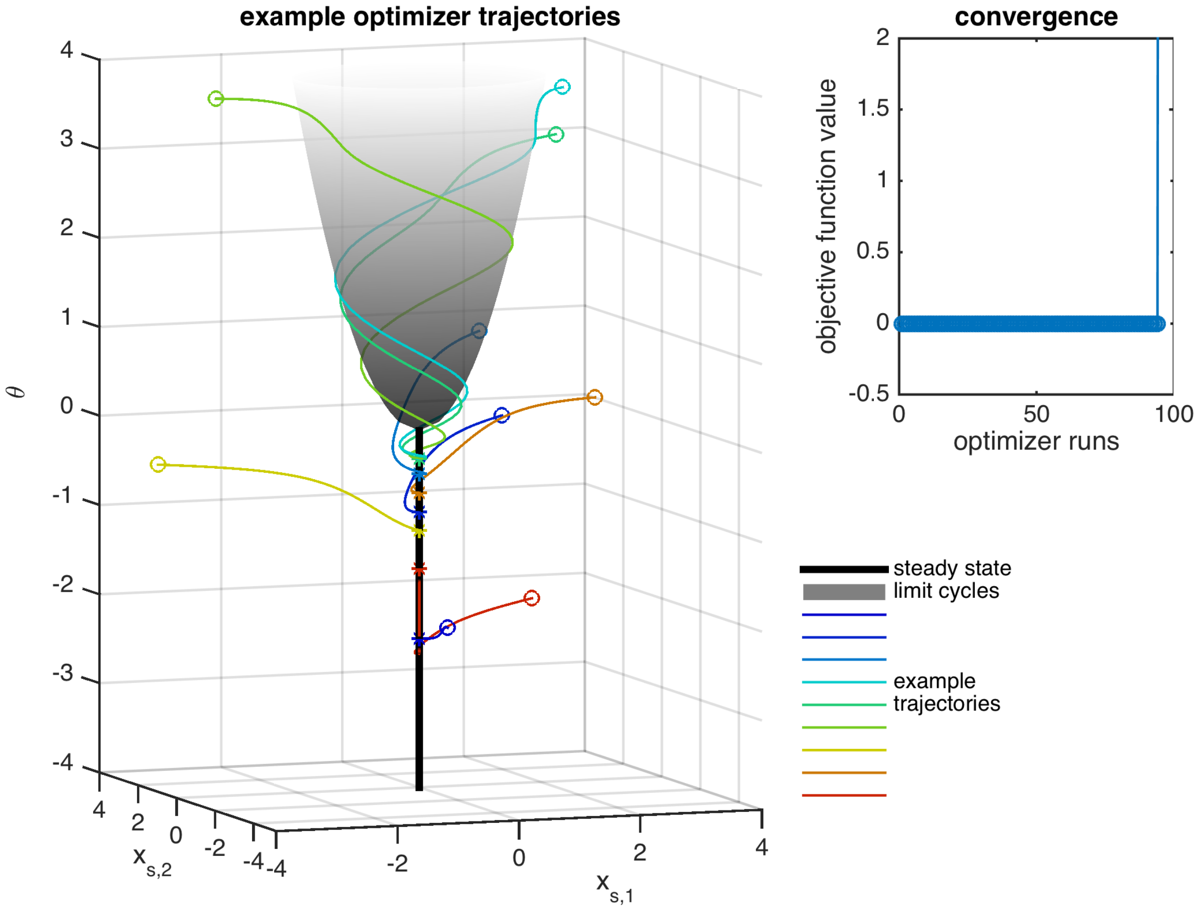

Supplement Figure 3: Evaluation of the simulation-based optimization method for the system with Hopf-bifurcation. (left) 10 example optimizer trajectories and (right) results of 100 local optimization runs.
