# Supplementary material for: Tailored parameter optimization methods for ordinary differential equation models with steady-state constraints
Source: BMC Syst Biol. 2016 Aug 22;10(1):80. doi: 10.1186/s12918-016-0319-7 (PMC4994295; doi:10.1186/s12918-016-0319-7)
Supplement: Additional file 2 — Code S1. This zip-file contains the MATLAB code used for the simulation example (conversion process) and the application examples (NGF-induced Erk signaling in primary sensory neurons and Raf/MEK/ERK signaling in HeLa cells after release from S-phase arrest) presented in the paper. We provide implementations for the hybrid optimization and simulation-based optimization methods, the models and the optimization. In addition to the implementation, also all data and result files (.mat,.csv) are included. (ZIP 25965 kb) [file 12918_2016_319_MOESM2_ESM.zip › Revision/readme.html]

Code to the paper „Tailored parameter optimization methods for ordinary differential equation models with steady-state constraints“ (in submission)

1. Main files

- *VFMO\_mod.m*

provides the gradient for the simulation-based optimization

- *sampleParameters.m*

samples parameters using latin hypercube sampling

- *Manifold\_opt.m*

is a multistart routine for simulation-based optimization

2. Examples

Simulation example:

- *ConversionReactionTimeSeries\_opt.m*

performs the optimization for the conversion process using the different methods saving the results in the Result folder.

Apllication example 1:

- *NGFIndErkSignaling\_opt.m*

performs the optimization for the NGF-induced ERK signaling using the different methods saving the results in the Result folder.

- *Supplement\_opt.m*

performs the parameter estimation for the NGF-induced optimization for different values of the retraction factor lambda.

Application example 2:

- *RafMekErk\_opt.m*

performs the optimization for the Raf/MEK/ERK signaling using the different methods saving the results in the Result folder.

- *modelComparison.m*

performs the parameter estimation for the Raf/MEK/ERK pathway model without feedback.

Supplementary information - hysteresis

- *hysteresis\_opt.m*

performs the optimization for the hysteresis system using the different methods saving the results.

Supplementary information - hopf

- *hopf\_opt.m*

performs the optimization for the hopf system using the different methods saving the results.

The parameters estimated by the simulation-based optimization using gradient descent and newton method are saved to the structs parameters\_SD and parameters\_NM, respectively.

The parameters estimated by hybrid optimization are saved to the struct parameters\_FMs.

The parameters estimated by constrained and unconstrained optimization are saved to the structs parameters\_FM and parameters\_FUa, respectively.

The evolution of parameters along the solver path and all other evolutions paths, which are saved by default, were deleted from the results of all optimization to decrease data size.

3. Figure generation

- *Figure\_ConversionReaction.m*

- *Figure\_ConversionReactionFMs.m*

create the Figure 3 and 4 used in the manuscript

- *Figure\_NGFInducedErkSignaling.m*

creates Figure 6

- *Figure\_RafMekErk.m*

creates Figure 8 and calculates the AIC and BIC for the Raf/MEK/ERK model with and without feedback.

- *Figure\_SupplementLambda.m*

creates Figure 1 from the supplement

- *figureHystersis.m*

creates Figure 2 from the supplement

- *figureHopf.m*

creates Figure 3 from the supplement
